# A Dopamine-Responsive Signal Transduction Controls Transcription of Salmonella enterica Serovar Typhimurium Virulence Genes

**DOI:** 10.1128/mBio.02772-18

**Published:** 2019-04-16

**Authors:** Dezhi Yang, Ying Kong, Wei Sun, Wei Kong, Yixin Shi

**Affiliations:** a School of Life Sciences, Arizona State University, Tempe, Arizona, USA; b Center for Immunotherapy, Vaccines, and Virotherapy, Biodesign Institute, Arizona State University, Tempe, Arizona, USA; c Department of Immunology & Microbial Disease, Albany Medical College, Albany, New York, USA; Institut Pasteur

**Keywords:** bacterial signal transduction, catecholamine neurotransmitter dopamine, MarR family virulence regulator EmrR, PhoP/PhoQ and SlyA feedforward loop, *Salmonella* pathogenicity island 2 (SPI-2)

## Abstract

We have shown that the ligand-responsive MarR family member SlyA plays an important role in transcription activation of multiple virulence genes in Salmonella enterica serovar Typhimurium by responding to guanosine tetraphosphate (ppGpp). Here, we demonstrate that another MarR family member, EmrR, is required for virulence of *S.* Typhimurium and another enteric bacterium, Yersinia pestis. EmrR is found to activate transcription of an array of virulence determinants, including *Salmonella* pathogenicity island 2 (SPI-2) genes and several divergent operons, which have been shown to be activated by SlyA and the PhoP/PhoQ two-component system. We studied the regulatory effect of EmrR on one of these genetic loci, i.e., the *pagC-pagD* divergent operon, and characterized a catecholamine neurotransmitter, dopamine, as an EmrR-sensed signal. Dopamine acts on EmrR to reduce its ability to bind to the target promoters, thus functioning as a negative signal to downregulate this EmrR-activated transcription. EmrR can bind to AT-rich sequences, which particularly overlap the SlyA and PhoP binding sites in the *pagC-pagD* divergent promoter. EmrR is a priming transcription regulator that binds its target promoters prior to successive transcription activators, by which it displaces universal silencer H-NS from these promoters and facilitates successive regulators to bind these regions. Regulation of the *Salmonella*-specific gene in Escherichia coli and Y. pestis reveals that EmrR-dependent regulation is conserved in enteric bacteria. These observations suggest that EmrR is a transcription activator to control the expression of virulence genes, including the SPI-2 genes. Dopamine can act on the EmrR-mediated signal transduction, thus downregulating expression of these virulence factors.

## Abstract (precis)

In this study, MarR family regulator EmrR is identified as a novel virulence factor of enteric bacteria, here exemplified by Salmonella enterica serovar Typhimurium and Yersinia pestis. EmrR exerts an essential effect as a transcription activator for expression of virulence determinants, including *Salmonella* pathogenicity island 2 genes and a set of horizontally acquired genetic loci that formed divergent operons. EmrR senses the neurotransmitter dopamine and is subsequently released from target promoters, resulting in downregulation of the virulence gene expression. Through this action on EmrR, dopamine can weaken *Salmonella* resistance against host defense mechanisms. This provides an explanation for the previous observation that dopamine inhibits bacterial infection in animal gastrointestinal tracts. Our findings provide evidence that this neurotransmitter can modulate bacterial gene expression through interaction with virulence regulator EmrR.

## INTRODUCTION

MarR family regulators play critical roles in multidrug resistance, oxidative stresses, catabolism of environmental aromatic compounds, antimicrobial peptide resistance, and virulence (1–7). Their members display significant diversity, responding to different signaling molecules and recognizing diverse DNA targets. First characterized in Escherichia coli, EmrR is a MarR family regulator encoded by the first gene of the *emrRAB* operon. It functions as a transcriptional repressor to control the synthesis of EmrAB, which interacts with the TolC outer membrane porin to construct a tripartite multidrug pump (8–11). Originally referred to as MprA, EmrR regulates the plasmid-borne *mcb* operon for microcin B17 synthesis (12). EmrR downregulates the *emrRAB* operon by binding to an inverted repeat sequence centered near the −10 region of its promoter (13). Accordingly, disruption of the *emrR* gene led to increased expression of EmrAB and elevated resistance to the antibiotic thiolactomycin (9). The presence of structurally unrelated substrates, including carbonyl cyanide *m*-chlorophenylhydrazone, carbonyl cyanide *p*-(trifluoromethoxy) phenylhydrazone, and 2,4-dinitrophenol derepress *emrRAB* expression by binding to EmrR and releasing it from the operon promoter, allowing expression of EmrAB (14). The EmrAB pump can then export these molecules (14). Except for the *emrRAB* operon and plasmid-borne *mcb* loci, it remains largely elusive which genetic loci are regulated by EmrR in E. coli or other enteric bacteria.

Although work in the area of MarR family-related regulatory mechanisms has been focused on their functions in transcriptional repression, cumulative evidence suggests that some MarR family regulators play important roles in transcriptional activation. For example, ExpG from the nitrogen-fixing soil bacterium Sinorhizobium meliloti activates transcription of three *exp* operons involved in the production of galactoglucan, an exopolysaccharide required for this organism to infect plant roots for nodule formation (15–17). In Salmonella enterica serovar Typhimurium, the MarR family SlyA protein has been characterized as a virulence regulator contributing to bacterial infection in part by activating hemolysin and flagellum production (18–20). This characterization was further supported by the observations that a master virulence regulator, i.e., the PhoP/PhoQ two-component system, activated transcription of the *slyA* gene (5, 21). When *Salmonella* experiences Mg^2+^ deprivation conditions, the sensor kinase PhoQ mediates phosphorylation of the response regulator PhoP (for a review, see reference 22), facilitating PhoP binding the target promoters and regulating transcription of these genes, which includes their operon, *phoPQ* and *slyA* (5, 21). Actually, the PhoP protein acts not only as the transcriptional activator of the *slyA* gene but also as one of two transcriptional activators (the other activator being SlyA) for a subset of horizontally acquired gene clusters, including several divergent operons (5, 23). This regulatory circuit, generally designated the feedforward regulatory loop (24), enables bacteria to regulate transcription by integrating different signals, i.e., the environmental low Mg^2+^ sensed by the PhoP/PhoQ system (25) and the cytoplasmic alarmone (ppGpp) sensed by SlyA (23). The regulators PhoP and SlyA recognized the target promoters via the PhoP binding site, TAAAT-N_6_-TAAAC (a PhoP box in the opposite strand) and the SlyA binding site, AATATT-N_10_-ATTATT (the SlyA box), respectively, in which they antagonized a global transcriptional silencer H-NS occupying their AT-rich binding sites (5, 23, 26, 27). This working model demonstrated that a MarR family regulator could participate in a regulatory network by coordinating with other signal response regulators to control genetic loci contributing to bacterial pathogenesis.

For the first time, we have elucidated that EmrR, a MarR family regulator sharing sequence similarity with SlyA, governs a signal transduction pathway in response to neurotransmitter dopamine and essential for virulence of enteric bacteria including *S.* Typhimurium and Yersinia pestis. We found that EmrR was able to activate transcription of *Salmonella* pathogenicity island 2 (SPI-2) genes and a set of horizontally acquired loci and characterized dopamine as a ligand that could act on EmrR to repress the EmrR-dependent transcription. We revealed that EmrR interacted with the AT-rich regions at its target promoters, by which it displaced a global transcription repressor H-NS from the promoters and enhanced other activators to bind to their binding sites. Therefore, our study illustrates a multifactorial regulatory cascade to control the expression of the *S.* Typhimurium virulence regulons in response to both positive and negative inputs.

## RESULTS

### MarR family member EmrR is a virulence regulator required for *S.* Typhimurium and Yersinia pestis infection.

**FIG 1:**
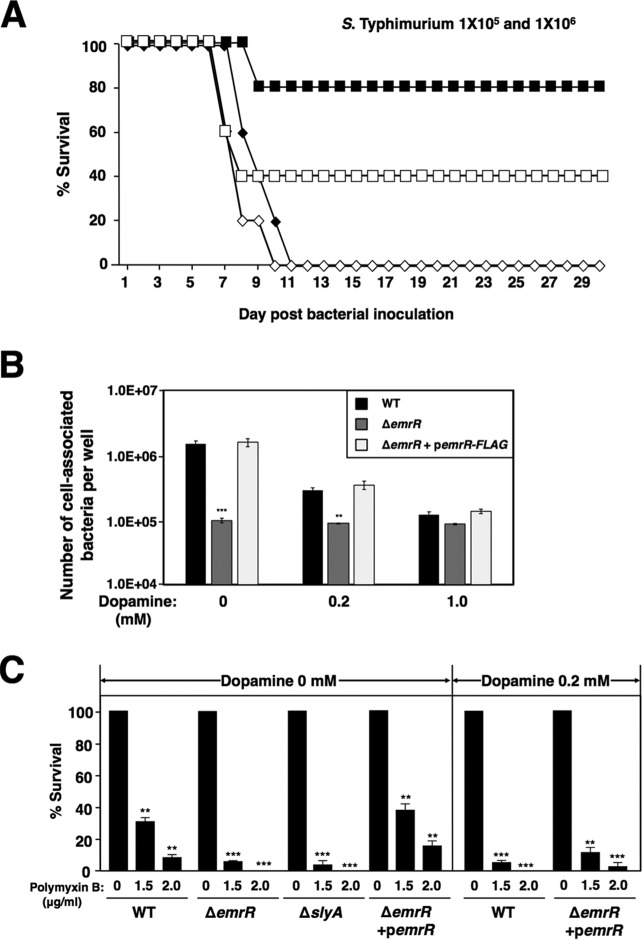
EmrR is an *S***.** Typhimurium virulence regulator in a mouse model and contributes to resistance to polymyxin B. (A) Survival of BALB/c mice (five mice in each group) administered orally with *Salmonella* wild-type (WT) strain 14028s (diamonds) and Δ*emrR* mutant (YS15776) (squares) at doses of 1 × 10^5^ CFU (solid symbols) and 1 × 10^6^ CFU (open symbols). WT versus Δ*emrR* (1 × 10^5^), *P < *0.01; WT versus Δ*emrR* (1 × 10^6^), *P < *0.01. (B) Intramacrophage growth levels of *Salmonella* wild-type strain 14028s (WT), Δ*emrR* mutant (YS15776), and Δ*emrR* mutant harboring complementing plasmid p*emrR-FLAG* (pYS2015) at a ratio of 10 bacteria per J774.2 cell (i.e., multiplicity of infection [MOI] of 10). Macrophages were mixed with bacterial cells, centrifuged, and then incubated for 20 min to permit phagocytosis. Macrophages were lysed after cultured in the medium containing dopamine at the indicated concentrations for 18 h and plated onto LB agar to determine the number of viable bacteria (average numbers of CFU of the intracellular bacteria for 2 h postinfection were 5.7E4 for WT, 5.1E4 for the Δ*emrR* mutant, and 6.3E4 for Δ*emrR* mutant plus p*emrR-FLAG*; see details in Materials and Methods). Values that are significantly different from the value for the WT group by *t* test are shown by asterisks as follows: ***, *P < *0.001; **, *P < *0.01. (C) Survival rates of *Salmonella* wild-type strain 14028s (WT), Δ*slyA* mutant (YS11068), Δ*emrR* mutant (YS15776), and Δ*emrR* mutant harboring p*emrR-FLAG* after the strains were challenged by antimicrobial peptide polymyxin B. Bacterial cultures were supplemented with dopamine at the indicated concentration or not supplemented with dopamine. Values that are significantly different from the value for the strain not treated with polymyxin B by *t* test are indicated by asterisks as follows: ***, *P < *0.001; **, *P < *0.01. Data in panels B and C are from three independent assays conducted in duplicate, and all values are means ± standard deviations.

Since MarR family regulator SlyA had been demonstrated as a virulence factor for *Salmonella* infection in a mouse model (18), we investigated other members of the MarR family for their possible roles in bacterial virulence. A mouse virulence analysis was carried out by using single in-frame deletion mutations at the genes encoding other MarR family members that shared homology with SlyA. While all the BALB/c mice inoculated orally with 10^5^ and 10^6^ CFU of wild-type ATCC 14028s (14028) cells died within 11 days, 80% and 40% of the mice that received an isogenic Δ*emrR* mutant survived 30 days postinoculation, respectively (Fig. 1A). In contrast, all mice died within 10 days after intraperitoneal administration of 10^2^ and 10^3^ CFU of the wild-type strain and Δ*emrR* mutant (unpublished results), suggesting that EmrR should be specifically required for the oral route of bacterial infection. A *Salmonella* survival assay in murine J774.2 macrophages showed that the number of CFU of the Δ*emrR* mutant was reduced ∼13-fold compared with that of the wild-type strain that survived within macrophages (Fig. 1B). The phenotype of the Δ*emrR* mutant was solely the result of a lack of the EmrR protein because the CFU in macrophages were recovered to a wild-type level in an Δ*emrR* mutant in which a FLAG-tagged *emrR* gene was expressed under the control of the P*_lac_* promoter from plasmid p*emrR-FLAG* (Fig. 1B). A significant feature often associated with *Salmonella* virulence was the inducible resistance to antimicrobial peptides (AMPs), many of which were regulated through specific virulence regulators (28–33). We investigated whether EmrR contributed to AMP resistance by challenging *Salmonella* strains with a cationic antimicrobial peptide, polymyxin B, and found that the Δ*emrR* mutant displayed a susceptible phenotype to polymyxin B in a manner similar to that of the Δ*slyA* mutant (Fig. 1C). The *emrR* mutation could be complemented in *trans* because the survival rates of the Δ*emrR* mutant harboring p*emrR-FLAG* was restored to wild-type levels when challenged by polymyxin B at different concentrations (Fig. 1C). Furthermore, we demonstrated that EmrR was a virulence factor not only in *Salmonella* but also in Yersinia pestis by carrying out a mouse virulence assay described previously (34). When Swiss Webster mice were injected subcutaneously with a wild-type strain KIM6+(pCD1Ap) and its isogenic Δ*emrR* mutant χ10064(pCD1Ap), the Δ*emrR* mutant was ∼1,000 times less virulent than its isogenic wild-type strain (see Fig. S1 in the supplemental material).

10.1128/mBio.02772-18.1 FIG S1 The Yersinia pestis *emrR* mutant is strongly attenuated in mice after subcutaneous infection. Survival of Swiss Webster mice injected subcutaneously (s.c.) with the wild-type strain (KIM6+ *vir*) at doses of 28 CFU (open diamonds) and 280 CFU (solid diamonds) and the Δ*emrR* mutant (χ10064 *vir*) at doses of 380 CFU (open squares) and 3,800 CFU (solid squares). Wild-type strain (28 CFU) versus Δ*emrR* mutant (380 CFU), *P < *0.01; wild-type strain (280 CFU) versus Δ*emrR* mutant (3,800 CFU), *P < *0.01. Download FIG S1, TIF file, 0.2 MB. Copyright © 2019 Yang et al. 2019 Yang et al. This content is distributed under the terms of the Creative Commons Attribution 4.0 International license.

### EmrR is required for transcription activation of *Salmonella* pathogenicity island 2 genes and virulence-related divergent operons.

**FIG 2:**
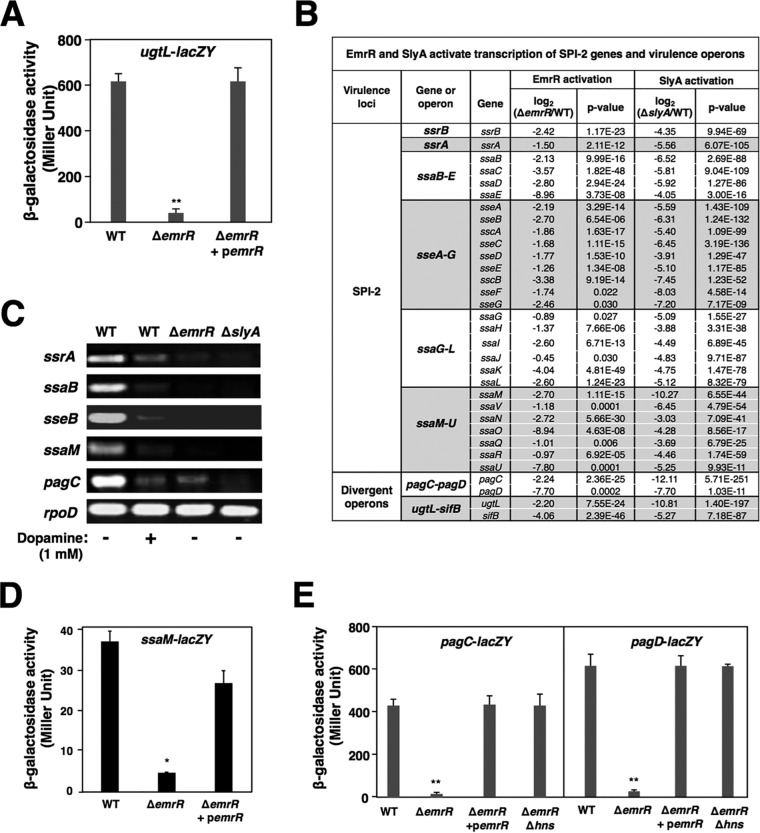
EmrR activates transcription of SPI-2 genes and the PhoP- and SlyA-activated divergent operons. (A) β-Galactosidase activity from a chromosomal *ugtL*-*lacZY* fusion was determined in the *S.* Typhimurium wild-type (WT) strain (YS15550), Δ*emrR* mutant (YS15708), and Δ*emrR* mutant harboring complementing plasmid p*emrR-FLAG* (pYS2015) grown in 0.01 mM (low) Mg^2+^ for 4 h. The value for the Δ*emrR* mutant was significantly different (*P < *0.01) by *t* test from the WT value as indicated (**). (B) RNA-Seq analysis shows downregulation of SPI-2 genes and horizontally acquired divergent operons in the Δ*emrR* mutant (YS15776) and Δ*slyA* mutant (YS11068) compared to the wild-type strain 14028s (WT) when grown in low Mg^2+^ for 4 h. (C) RT-PCR analysis for the mRNA levels of transcripts from four SPI-2 genes and also the *pagC* gene in the wild-type strain 14028s (WT), Δ*emrR* mutant (YS15776), and Δ*slyA* mutant (YS11068) grown in low Mg^2+^ with or without 1 mM dopamine and from the Δ*emrR* mutant (YS15776) and Δ*slyA* mutant (YS11068) grown in low Mg^2+^. The constitutively transcribed *rpoD* gene was used to indicate similar amounts of total RNAs. The DNA fragment was amplified using the primers listed in Table S2 and separated in an agarose gel. Dopamine was added to the required bacterial culture at a final concentration of 1 mM. (D) β-Galactosidase activity from a chromosomal *ssaM*-*lacZY* fusion was determined in the wild-type strain (YS18490), Δ*emrR* mutant (YS18520), and Δ*emrR* mutant harboring p*emrR-FLAG* grown in low Mg^2+^ supplemented with 0.2 mM IPTG for 4 h. *, *P < *0.05 versus WT value by *t* test. (E) β-Galactosidase activity from respective chromosomal *pagC*-*lacZY* and *pagD*-*lacZY* fusions was determined in the WT strains (YS11644 and YS12000), Δ*emrR* mutants (YS14827 and YS17260), Δ*emrR* mutants harboring p*emrR-FLAG*, and Δ*emrR* Δ*hns* double mutants (YS15468 and YS17261) cultured in low Mg^2+^ for 4 h. **, P < 0.01 versus WT blue by *t* test. Data in panels A, D, and E are from three independent assays conducted in duplicate, and all values are means plus standard deviations.

As shown previously, the PhoP/PhoQ two-component system built a feedforward loop in association with SlyA to activate transcription of a polymyxin B resistance gene, *ugtL*, in a PhoP-activating condition (0.01 mM Mg^2+^; referred to as low Mg^2+^) (5, 23). Actually, EmrR was able to activate *ugtL* transcription in a low-Mg^2+^ condition in which the β-galactosidase activity in a strain harboring the chromosomal *ugtL*-*lacZY* fusion was 15.5-fold higher than that in its isogenic Δ*emrR* mutant (Fig. 2A). The deficient *ugtL* expression in the Δ*emrR* mutant was merely caused by an absence of this activator and could be rescued by an EmrR protein produced from plasmid p*emrR-FLAG*. The *ugtL* activation was specifically dependent on SlyA and EmrR, but not on other known MarR family regulators, since a deletion at the coding region of the *marR*, *STM1100*, *STM1547*, or *STM2920* gene did not affect *ugtL* transcription (Fig. S2A). An RNA-Seq analysis was carried out to profile more *Salmonella* genetic loci regulated by EmrR under low-Mg^2+^ growth (Table S3). The RNA-Seq data were first validated by increased RNA levels of the *emrA* and *emrB* genes in the Δ*emrR* mutant (5.7- and 5.9-fold, respectively; Table S3), since transcription of these genes was shown to be repressed by EmrR previously (9). Strikingly, almost all genetic loci of *Salmonella* pathogenicity island 2 (SPI-2) were detected as EmrR-activated genes, since their mRNA levels were significantly higher in the wild-type strain than in the Δ*emrR* mutant (Table S3; also summarized in Fig. 2B). Consistent with the previous observation that SlyA activated transcription of SPI-2 genes (35), RNA-Seq analysis also showed that the mRNA levels of these SPI-2 genes in the wild-type strain were higher than those in the Δ*slyA* mutant (Table S4; also summarized in Fig. 2B). We further confirmed the RNA-Seq results by a reverse transcription-PCR (RT-PCR) analysis which revealed higher mRNA levels of *ssrA*, *ssaB*, *sseB*, and *ssaM* genes from different SPI-2 operons in the wild-type strain than in the Δ*emrR* and Δ*slyA* mutants, respectively (Fig. 2C) and with a β-galactosidase assay of reporter strains carrying a chromosomal *ssaM*-*lacZY* fusion in which the activity in the Δ*emrR* mutant was 8-fold lower than that in the wild-type strain (Fig. 2D). Consistently, deficient *ssaM* expression in the Δ*emrR* mutant could be restored by a heterologous *emrR* expression from plasmid p*emrR-FLAG* (Fig. 2D). It was shown that the PhoP/PhoQ and SlyA feedforward loop simultaneously activated transcription of a set of divergent operons, including *ugtL*-*sifB* and *pagC*-*pagD* (5, 22). Concomitantly, RNA-Seq analysis showed that mRNA levels of *ugtL*, *sifB*, *pagC*, and *pagD* in the Δ*emrR* mutant strain were significantly reduced compared to those in the wild-type strain (Table S3; also see Fig. 2B). The EmrR-dependent regulation of this class of genetic loci was further confirmed by a β-galactosidase assay of reporter strains carrying chromosomal *pagC*-*lacZY* and *pagD*-*lacZY* fusions in which activities in the Δ*emrR* mutants were 25.2- and 22.5-fold reduced, respectively, compared to those in their isogenic wild-type strains grown in low Mg^2+^ (Fig. 2E). Furthermore, the deficient expression of this divergent operon in the Δ*emrR* mutant could be restored by heterologous *emrR* expression from plasmid p*emrR-FLAG* (Fig. 2E). We found that deletion of the transcriptional silencer *hns* gene could restore both *pagC* and *pagD* transcription in the Δ*emrR* mutant to wild-type levels (Fig. 2E), suggesting that EmrR should function as an antirepressor to antagonize the H-NS effect at the *pagC*-*pagD* divergent promoter in a manner comparable to that of PhoP and SlyA (27). In contrast, EmrR did not exert an effect on transcription merely dependent on the PhoP/PhoQ system because transcription of two PhoP-activated genes, *pcgL* and *STM3595* (36), remained at similar levels in wild-type and Δ*emrR* strains (Fig. S3A). Therefore, we demonstrated that EmrR could function as a transcription activator to enhance transcription of the SPI-2 genes and those stimulated by both PhoP and SlyA regulators.

10.1128/mBio.02772-18.2 FIG S2 Characterization of MarR family member EmrR as a transcription activator of the PhoP- and SlyA-dependent genes and its negative signal molecule in *S*. Typhimurium. (A) β-Galactosidase activity from a chromosomal *ugtL*-*lacZY* fusion was determined in the wild-type strain (WT) (YS15550), Δ*slyA* mutant (YS11610), Δ*emrR* mutant (YS15708), Δ*marR* mutant (YS18663), Δ*STM1100* mutant (YS18662), Δ*STM1547* mutant (YS18660), and Δ*STM2920* mutant (YS18661) grown in low Mg^2+^ for 4 h. ***, *P < *0.001 versus WT value by *t* test. (B) β-Galactosidase activity from the *pagC*-*lacZY* strain (YS11644) grown in low Mg^2+^ supplemented with phenolic compounds for 4 h was determined. The first bar labeled None represents the activity from the culture without any tested compound. **, *P < *0.01 versus the None value by *t* test. (C) Alignment of the amino acid sequences of EmrR and SlyA from *S*. Typhimurium. The highlighted sequences formed the α3, α4, and β wing regions revealed previously (40). Sequences producing a significant alignment with an E value of 6E−06 are shown. Data in panels A and B are from three independent assays conducted in duplicate, and all values are means ± standard deviations. Download FIG S2, TIF file, 0.8 MB. Copyright © 2019 Yang et al. 2019 Yang et al. This content is distributed under the terms of the Creative Commons Attribution 4.0 International license.

10.1128/mBio.02772-18.3 FIG S3 EmrR is not a transcription activator of the PhoP-dependent genes. (A) β-Galactosidase activity from chromosomal *pcgL*-*lacZY* and *STM3595*-*lacZY* fusions was determined in wild-type strains (WT) (YS10382 and YS11620) and Δ*emrR* mutants (YS15733 and YS15732) grown in low Mg^2+^ for 4 h. *P > *0.05 (not significant) versus WT value by *t* test. (B) β-Galactosidase activity was determined in the *pcgL*-*lacZY* strain (YS10382) and *STM3595*-*lacZY* strain (YS11620) in low Mg^2+^ supplemented with 2 mM dopamine or without dopamine for 4 h. *P > *0.05 (not significant) versus the WT value by *t* test. (C) Immunoblot analysis for determination of the levels of the SlyA-FLAG protein produced in the *slyA-FLAG* strain (YS10075) grown in low Mg^2+^ and overproduced in the *pagC-lacZY* Δ*emrR* Δ*slyA* mutant (YS15533) harboring p*slyA*-*FLAG* (pYS1109) grown in low Mg^2+^ with 1 mM IPTG for 4 h. (D) Immunoblot analysis for determination of the levels of EmrR-FLAG protein overproduced in *emrR-FLAG* strain (YS16035) grown in low Mg^2+^ and overproduced in *pagC-lacZY* Δ*emrR* Δ*slyA* mutant (YS15533) harboring p*emrR*-*FLAG* (pYS2015) grown in low Mg^2+^ with1 mM IPTG or without IPTG for 4 h. Data in panels A and B are from an assay conducted in triplicate. In panels C and D, the *corA-FLAG* strain (YS11477) was used as a control grown under the same conditions. Download FIG S3, TIF file, 0.3 MB. Copyright © 2019 Yang et al. 2019 Yang et al. This content is distributed under the terms of the Creative Commons Attribution 4.0 International license.

### The catecholamine neurotransmitter dopamine is a signal molecule to abrogate EmrR-activated transcription.

**FIG 3:**
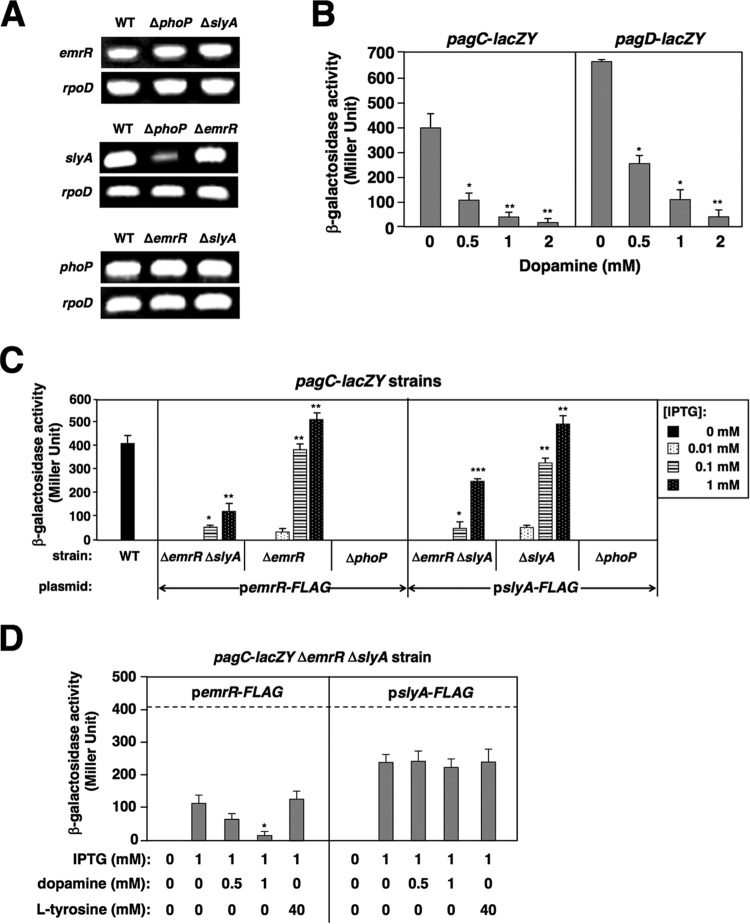
EmrR responds to dopamine specifically to repress transcription of its target genes in *S.* Typhimurium. (A) RT-PCR analysis to determine the levels of *phoP*, *slyA*, and *emrR* mRNAs in wild-type (WT) strain 14028s, Δ*phoP* mutant (YS11590), Δ*slyA* mutant (YS11068), and Δ*emrR* mutant (YS15776) grown in low Mg^2+^ for 4 h. (B) β-Galactosidase activity from respective chromosomal *pagC*-*lacZY* and *pagD*-*lacZY* fusion was determined in wild-type strains (YS11644 and YS12000) cultured in low Mg^2+^ for 4 h with dopamine at indicated concentrations. Values that are significantly different from growth with 0 mM dopamine by *t* test are indicated by asterisks as follows: **, *P < *0.01; *, *P < *0.05. (C) β-Galactosidase activity was determined in the wild-type (WT) *pagC*-*lacZY* strain (YS11644), Δ*emrR* Δ*slyA* mutant (YS15533), Δ*emrR* mutant (YS14827), and Δ*phoP* mutant (YS11782) harboring complementing plasmid p*emrR-FLAG* (pYS2015), or p*slyA-FLAG* (pYS1109) grown in low Mg^2+^ with the indicated IPTG concentrations for 4 h. Values that are significantly different from growth with 0 mM IPTG by *t* test are indicated by asterisks as follows: ***, *P < *0.001; **, *P < *0.01; *, *P < *0.05. (D) β-Galactosidase activity was determined in the *pagC*-*lacZY* Δ*emrR* Δ*slyA* mutant (YS15533) harboring p*emrR-FLAG* or p*slyA-FLAG* grown in low Mg^2+^ supplemented with 1 mM IPTG or without IPTG as well as dopamine at indicated concentrations for 4 h. The dashed line indicates the β-galactosidase level in the wild-type *pagC*-*lacZY* strain (YS11644). The value that was significantly different (*P < *0.05) from the value for growth with 1 mM IPTG, 0 mM IPTG, and 0 mM l-tyrosine by *t* test is indicated by an asterisk. Data in panels B to D are from three independent assays conducted in duplicate, and all values are means plus standard deviations.

In contrast to PhoP-activated *slyA* transcription (5, 21), transcription of the *emrR* gene was not regulated by either PhoP/PhoQ system or SlyA, since the levels of *emrR* transcripts were similar in the wild-type, Δ*phoP*, and Δ*slyA* strains (Fig. 3A, top panel). Also, the *slyA* and *phoP* mRNA levels were similar in the wild-type and Δ*emrR* strains (Fig. 3A, middle and bottom panels), which ruled out the possibility that EmrR could stimulate *slyA* and *phoPQ* transcription. The result also confirmed that *slyA* transcription was activated by the PhoP/PhoQ system (5) because the *slyA* mRNA level in the Δ*phoP* mutant was significantly reduced than that in the wild-type strain (Fig. 3A, middle panel). We postulate that the ligand-responsive regulator EmrR should also be able to respond to a specific signal molecule since most MarR family regulators bind small-molecule ligands, often phenolic compounds (37, 38). We chose some bioactive phenolic compounds (39) and investigated their effects on transcription of EmrR-regulated *pagC* gene on the premise that EmrR could sense one of these molecules. Among some 58 chosen phenolic compounds, we found that catecholamine neurotransmitter dopamine caused significant repression of *pagC* transcription (Fig. S2B). Consistently, β-galactosidase activity in the *pagC*-*lacZY* strain grown in low Mg^2+^ was reduced by supplementing dopamine in a concentration-dependent manner (Fig. 3B). Apparently, dopamine exerted its inhibitory effect on transcription of the *pagC*-*pagD* divergent operon since it could also reduce the β-galactosidase activity from a *pagD*-*lacZY* strain in a concentration-dependent manner (Fig. 3B). On the other hand, dopamine could not act on the PhoP/PhoQ system because the PhoP-activated transcription of the *pcgL* and *STM3595* genes remained unchanged under the conditions with 2 mM dopamine or without dopamine (Fig. S3B). Furthermore, RT-PCR analysis showed that dopamine could reduce mRNA levels of the tested SPI-2 genes in low Mg^2+^ (Fig. 2C). Concomitantly, dopamine reduced the survival rates of the wild-type strain living within macrophages and challenged by polymyxin B in a concentration-dependent manner, respectively (Fig. 1B and 1C). In this study, our results also showed that dopamine exerted a significant negative effect only in the presence of EmrR but could not further lower the survival rate of the Δ*emrR* mutant within macrophages (Fig. 1B). A sequence alignment analysis indicated that EmrR and SlyA exhibited ∼30% amino acid sequence identity (Fig. S2C) and that many of the conserved residues were clustered in the helices α3 and α4 as well as the β wing for DNA recognition (40). As a matter of fact, our previous study revealed that SlyA, at a high level that could not be attained physiologically in a wild-type cell, could stimulate deficient transcription of both *pagC* and *pagD* genes caused by an absence of the positive signal, i.e., alarmine-guanosine pentaphosphate (23). We found that SlyA when overproduced to 4.6-fold higher than the wild-type level in a Δ*emrR* Δ*slyA* mutant harboring plasmid p*slyA-FLAG* (36) (Fig. S3C), stimulated *pagC* transcription even in the absence of EmrR to 61% of the wild-type level (Fig. 3C). Likewise, EmrR at 3.0-fold higher than the wild-type level (Fig. S3D) was able to stimulate the *pagC* transcription to 29% of the wild-type level in the double mutant harboring p*emrR-FLAG* in which SlyA was absent (Fig. 3C). Therefore, we took advantage of this partially overlapped function to investigate whether EmrR could be the responsive regulator to dopamine. We found that the EmrR-stimulated *pagC* transcription in the Δ*emrR* Δ*slyA* mutant carrying plasmid p*emrR-FLAG* was 7.1-fold reduced by 1 mM dopamine, whereas the SlyA-stimulated transcription in this mutant carrying p*slyA-FLAG* was not influenced (Fig. 3D). l-Tyrosine, a precursor for biosynthesis of dopamine (41), did not affect the EmrR-stimulated *pagC* transcription even at a concentration as high as 40 mM (Fig. 3D). Taken together, we postulate that dopamine can serve as a signal molecule to act on EmrR and subsequently modulate *S.* Typhimurium gene regulation.

### EmrR binds to the *pagC*-*pagD* promoter regions at both PhoP and SlyA recognition sequences.

**FIG 4:**
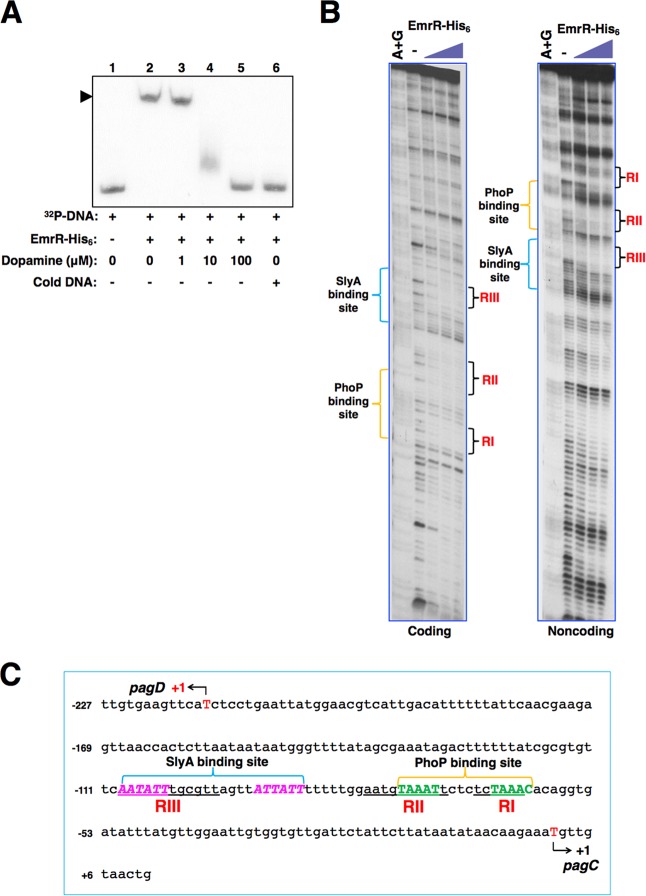
EmrR binding sites in an *S.* Typhimurium PhoP- and SlyA-activated promoter region. (A) EMSA for EmrR binding to the *pagC*-*pagD* promoter region. A ^32^P-labeled *pagC*-*pagD* promoter fragment, including the SlyA box and PhoP box sequences, was incubated with EmrR-His_6_ protein. Dopamine at the indicated concentrations and cold PCR products were added to release EmrR protein and competed for the binding of EmrR protein from the^32^P-labeled DNA fragment, respectively. *Arrow* indicates the shifted DNA fragment. (B) DNase I footprinting analysis of the *pagC*-*pagD* promoter with ^32^P-labeled probes for the coding and noncoding strands and increasing amounts of EmrR-His_6_ protein (0 [-], 50, 100, and 200 pmol [concentration indicated by the height of the purple triangles]). Products were subjected to polyacrylamide DNA sequencing electrophoresis, and the bands were detected by autoradiography. The brackets to the right of each gel image indicate the positions of the three EmrR-protected DNA regions (labeled RI, RII, and RIII). The brackets to the left of each gel image indicate the positions of the SlyA binding site and PhoP binding site characterized previously (23). Lane A+G corresponds to the Maxam and Gilbert A+G reaction ladder. (C) DNA sequence of the *pagC-pagD* promoter region. The three EmrR-protected regions (RI, RII, and RIII) characterized in panel B are underlined. The regions that interact with SlyA and PhoP are shown by blue and yellow brackets, respectively. The SlyA box sequence (pink bold italic capital letters) and the reversed PhoP box sequence (green bold capital letters) are indicated. The transcription initiation sites (+1) of *pagD* and *pagC* genes are indicated by red capital letters with arrows. Numbering is from +1 of *pagC*.

We conducted an electrophoretic mobility shift assay (EMSA) using a *pagC*-*pagD* promoter fragment which possesses both PhoP and SlyA binding sequences (namely, PhoP and SlyA boxes; see reference 23) to investigate EmrR binding to the target promoters. We found that an EmrR-His_6_ protein shifted this promoter fragment on the gel (Fig. 4A, lane 2). In this *in vitro* system, dopamine alone was sufficient to reduce the affinity of a pure EmrR protein to the promoter DNA fragment in a concentration-dependent manner (Fig. 4A, lanes 3 to 5). In contrast, dopamine, at a high concentration that actually eliminated EmrR-promoter interaction (Fig. 4A, lane 5), was unable to change the binding of SlyA-His_6_ protein to the same *pagC*-*pagD* promoter fragment (Fig. S4). This specific *in vitro* interaction between dopamine and the EmrR protein suggested the possibility that dopamine was a ligand molecule to act on EmrR. A DNase I footprinting analysis confirmed the promoter binding of EmrR and revealed three major EmrR-protected AT-rich regions, marked RI, RII, and RIII (Fig. 4B). We found that the RI, RII, and RIII regions were localized between −67 and −62, −80 and −71, and −109 and −98 nucleotide sequences upstream of the *pagC* transcription start site, which meanwhile corresponded to the locations between −153 and −148, −144 and −13, and −117 and −106 nucleotide sequences upstream of the *pagD* transcription start site, respectively (Fig. 4C). Actually, the RI and RII sequences overlapped the PhoP box, TAAAT-(6 nt)-TAAAC, and the RIII sequence partially overlapped the SlyA box, AATATT-(10 nt)-ATTATT (Fig. 4C), thus elucidating that EmrR shared its binding sites with PhoP and SlyA simultaneously. However, there were no consensus sequences that could be found from these binding sites except for their AT-rich feature. It appeared that EmrR might also interact, however weakly, with other AT-rich regions adjacent to RI and RIII sites (Fig. 4B). We reasoned that EmrR might recognize AT-rich regions rather than a specific consensus sequence since RI, RII, and RIII were neither homologous to each other (Fig. 4C) nor to an EmrR binding site characterized previously from the E. coli *emr* promoter (13).

10.1128/mBio.02772-18.4 FIG S4 Dopamine does not influence SlyA binding to the *S*. Typhimurium *pagC-pagD* promoter region. EMSA assay was carried out using 1 pmol of the same ^32^P-labeled *pagC*-*pagD* promoter fragment (from Fig. 4A) and 50 pmol of SlyA-His_6_ protein. Dopamine at 100 μM and cold PCR product (20 nM) were added to determine their effects on SlyA binding to the promoter fragment. The arrow indicates the shifted DNA fragment. Download FIG S4, TIF file, 0.1 MB. Copyright © 2019 Yang et al. 2019 Yang et al. This content is distributed under the terms of the Creative Commons Attribution 4.0 International license.

### EmrR acts as a priming regulator to facilitate binding of successive regulators to the target promoters.

**FIG 5:**
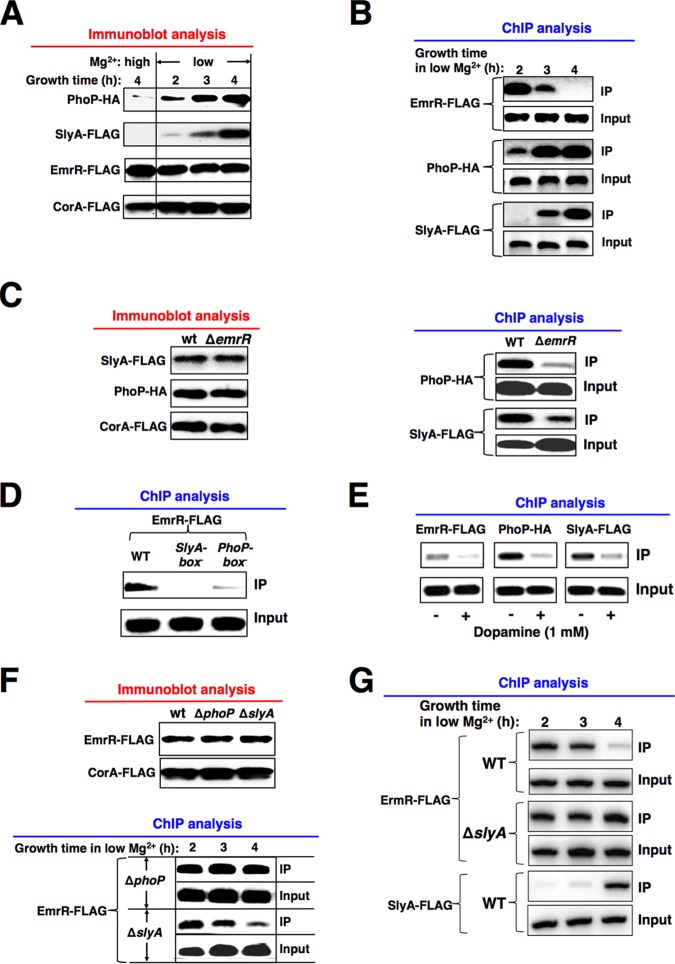
EmrR interacts with the promoter region to facilitate binding of other regulators in *S.* Typhimurium. (A) Immunoblot analysis for determination of the levels of EmrR-FLAG, PhoP-HA, and SlyA-FLAG proteins from the *phoP-HA* strain (YS11591), *slyA-FLAG* strain (YS10075), and *emrR-FLAG* strain (YS16035) grown in high (10 mM) or low Mg^2+^ for the indicated growth time. The level of CorA-FLAG protein from the *corA-FLAG* strain (YS11477) was used as an Mg^2+^-independent control. (B) ChIP analysis for *in vivo* binding of EmrR-FLAG, PhoP-HA, and SlyA-FLAG proteins to the *pagC-pagD* promoter region in the *emrR-FLAG* strain, *phoP-HA* strain, and *slyA-FLAG* strain grown in low Mg^2+^ for 2 to 4 h, respectively. PCRs were conducted using DNA templates in immunoprecipitated samples (IP) and total lysates (Input). (C, left) Immunoblot analysis of the total levels of SlyA-FLAG and PhoP-HA proteins in *slyA-FLAG* and *phoP-HA* strains, WT strains (YS10075 and YS11591) and their isogenic Δ*emrR* mutants (YS15602 and YS15601) grown in low Mg^2+^ for 4 h). (Right) ChIP analysis for *in vivo* promoter binding of SlyA-FLAG and PhoP-HA in these bacterial cells. (D) ChIP analysis for the *in vivo* binding of the EmrR-FLAG protein to the *pagC-pagD* promoter region in the *emrR-FLAG* strain, WT strain (YS16035) and the isogenic *PhoP-box_pagC-pagD_* mutant (YS17214), and *SlyA-box_pagC-pagD_* mutant (YS17213) grown in low Mg^2+^ for 3 h. (E) ChIP analysis for *in vivo* binding of EmrR-FLAG, PhoP-HA, and SlyA-FLAG proteins to the *pagC-pagD* promoter region in the *emrR-FLAG* strain (YS16035), *phoP-HA* strain (YS11591), and *slyA-FLAG* strain (YS10075) grown in low Mg^2+^ supplemented with 1 mM dopamine or without dopamine for 3 h. (F, top) Immunoblot analysis of the total levels of EmrR-FLAG protein in the *emrR-FLAG* strain, WT strain (YS16035) and their isogenic Δ*phoP* and Δ*slyA* mutants (YS15538 and YS15537) grown in low Mg^2+^ for 4 h. (Bottom) ChIP analysis for *in vivo* binding of EmrR-FLAG protein to the *pagC-pagD* promoter region in the Δ*phoP* mutant (YS15538) and Δ*slyA* mutant (YS15537) grown in low Mg^2+^ for 2 to 4 h. (G) ChIP analysis for *in vivo* binding of EmrR-FLAG and SlyA-FLAG proteins to the *ssrA* promoter region in the *emrR-FLAG* strain, WT strain (YS16035) and Δ*slyA* mutant (YS15537), and the *slyA-FLAG* strain and WT strain (YS10075) grown in low Mg^2+^ for 2 to 4 h. PCRs were conducted using DNA templates in immunoprecipitated samples (IP) and total lysates (Input). Data in panels A to F are from one of three independent assays.

In contrast with increased levels of PhoP and SlyA and continuous activation of *pagC-pagD* transcription in a time-dependent manner, the level of EmrR remained unchanged during the growth of bacterial cells in low Mg^2+^ for 4 h (Fig. 5A and Fig. S5A). We performed chromatin immunoprecipitation analysis (ChIP) to determine the *in vivo* interaction of EmrR to the *pagC*-*pagD* promoter in the *emrR-FLAG* strain and observed that the protein level of promoter-bound EmrR gradually decreased during the low-Mg^2+^ growth (Fig. 5B). Actually, the promoter decreased binding to EmrR and simultaneously increased binding to PhoP and SlyA in a time-dependent manner since the amounts of the promoter fragments pulled down by PhoP-HA and SlyA-FLAG proteins were proportional to their protein levels in the *phoP*-*HA slyA*-*FLAG* strain (Fig. 5A and B). Immunoblot analysis showed that the total levels of PhoP-HA and SlyA-FLAG proteins were similar in this *phoP*-*HA slyA*-*FLAG* strain and its isogenic Δ*emrR* mutant (Fig. 5C, left panel). However, the levels of PhoP-HA and SlyA-FLAG proteins that bound to the promoter were much lower in the Δ*emrR* mutant than in the wild-type strain (Fig. 5C, right panel), indicating that the role of EmrR was to enhance the binding of PhoP and SlyA to the promoter. In accord with the *in vitro* binding result (Fig. 4B), EmrR interacted specifically with the SlyA and PhoP boxes of the *pagC*-*pagD* promoter, since nucleotide substitutions at either sequence, which were referred to as *SlyA-box*^-^ or *PhoP-box*^-^ and shown to cause downregulation of the *pagC*-*pagD* transcription previously (23), mostly abrogated the promoter-EmrR interaction *in vivo* (Fig. 5D). Consistent with its effect *in vitro* (Fig. 4A), ChIP analysis showed that dopamine added to the culture of *emrR*-*FLAG* strain caused a reduction in EmrR binding to the *pagC*-*pagD* promoter *in vivo* (Fig. 5E). Simultaneously, dopamine also caused a reduction in the binding ability of PhoP-HA and SlyA-FLAG proteins to the promoter *in vivo* (Fig. 5E), thus providing further evidence that EmrR enhanced the binding of PhoP and SlyA to the target promoter. It is worth noting that the bacterial cells cultured for 3 h were used to carry out the ChIP analyses in Fig. 5D and E, since more EmrR-FLAG proteins were found to bind to the *pagC-pagD* promoter in the wild-type strain (Fig. 5B). Under this growth condition, a reduced level of the promoter-bound EmrR caused by a mutation of the promoter sequence or supplementing with dopamine could be more accurately compared. Mutation of the *slyA* and *phoP* genes did not affect the expression of EmrR, since its total levels were similar in wild-type and mutant cells grown in low Mg^2+^ for 4 h (Fig. 5F, top panel). However, the bound EmrR could not be dissociated from the *pagC*-*pagD* promoter in the Δ*phoP* mutant, and also more EmrR proteins remained bound to the promoter in the Δ*slyA* mutant than in the wild-type strain (Fig. 5F, bottom panel). We postulate that EmrR should be displaced from the promoter by PhoP and SlyA in a cooperative manner, because the absence of either regulator caused more EmrR bound to the promoter. Importantly, similar to the role in the *pagC-pagD* promoter, EmrR was also displaced by SlyA from the *ssrA* promoter in SPI-2 because the level of promoter-bound EmrR and SlyA decreased and increased simultaneously in a time-dependent manner during the low-Mg^2+^ growth (Fig. 5G). Subsequently, more EmrR proteins remained bound to this promoter in the Δ*slyA* mutant than in the wild-type strain (Fig. 5G), thus reconfirming that SlyA could displace EmrR from this promoter.

10.1128/mBio.02772-18.5 FIG S5 Examination of transcription of the *pagC*-*pagD* operon in *S*. Typhimurium and E. coli**.** (A) β-Galactosidase activity from *pagC*-*lacZY* (YS11644) and *pagD*-*lacZY* (YS12000) strains grown in high or low Mg^2+^ was determined at indicated times. (B) β-Galactosidase activity from wild-type E. coli strain BW25113 (WT) and the isogenic Δ*slyA* mutant (YS14989) and Δ*emrR* mutant (YS14986) carrying pYS1031 and a compatible plasmid pUHE21-2*lacI*^q^ (i.e., vector) or p*emrR-FLAG* (pYS2015) grown in low Mg^2+^ supplemented with 1 mM IPTG and with 1 mM dopamine or without dopamine for 4 h. Values that are significantly different from the WT value by *t* test are indicated by asterisks as follows: ***, *P < *0.001; *, *P < *0.05. Data are from three independent assays conducted in duplicate, and all values are means ± standard deviations. Download FIG S5, TIF file, 0.3 MB. Copyright © 2019 Yang et al. 2019 Yang et al. This content is distributed under the terms of the Creative Commons Attribution 4.0 International license.

### EmrR controls the PhoP/PhoQ and SlyA feedforward regulatory loop in many enteric bacteria.

**FIG 6:**
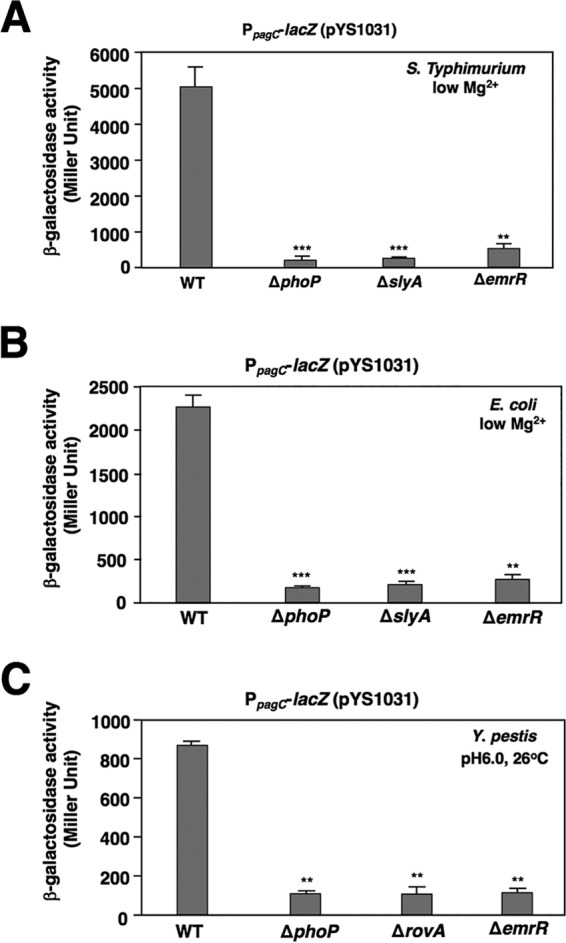
EmrR conserves transcription activation by the PhoP and SlyA feedforward loop in various enteric bacteria. (A) β-Galactosidase activity from P*_pagC_*-*lacZ* transcriptional fusion cloned in a plasmid (pYS1031) was determined in *S.* Typhimurium wild-type strain 14028s (WT), Δ*phoP* mutant (YS11590), Δ*slyA* mutant (YS11068), and Δ*emrR* mutant (YS15776) grown in low Mg^2+^ for 4 h. Values that are significantly different from the WT value by *t* test are indicated by asterisks as follows: ***, *P < *0.001; **, *P < *0.01. (B) β-Galactosidase activity from the P*_pagC_*-*lacZ* transcriptional fusion (pYS1031) was determined in the wild-type E. coli strain BW25113 (WT) and the isogenic Δ*phoP* mutant (YS14985), Δ*slyA* mutant (YS14989), and Δ*emrR* mutant (YS14986) grown in low Mg^2+^ for 4 h. Values that are significantly different from the WT value by *t* test are indicated by asterisks as follows: ***, *P < *0.001; **, *P < *0.01. (C) β-Galactosidase activity from P*_pagC_*-*lacZ* transcriptional fusion (pYS1031) was determined in the wild-type Y. pestis strain Kim6+ (WT) and the isogenic Δ*phoP* mutant (χ10038), Δ*rovA* mutant (χ10046), and Δ*emrR* mutant (χ10064) grown in low Mg^2+^ at pH 6.0 at 26°C for 4 h. Values that are significantly different (*P < *0.01) from the WT value by *t* test are indicated (**). Data in panels A to C are from three independent assays conducted in duplicate, and all values are means plus standard deviations.

Sequence alignment analysis indicated that *S*. Typhimurium PhoP, SlyA, and EmrR were conserved in many enteric bacteria and specifically that the amino acid sequences revealed 93%, 79%, and 89% identity to their orthologs in E. coli and 75%, 93%, and 80% identity to their orthologs in Yersinia pestis. However, genetic loci regulated by the feedforward loop via these regulators remain elusive in these organisms. Thus, we investigated whether these orthologs could constitute the same regulatory network by using a reporter plasmid, pYS1031, which harbored a *lacZ* fusion with a *pagC* promoter fragment, including the SlyA and PhoP boxes (23). The cloned promoter fragment in this plasmid was sufficient to render not only a PhoP- and SlyA-dependent regulation (23) but also an EmrR-dependent regulation, since β-galactosidase activity in the *Salmonella* Δ*emrR* mutant harboring pYS1031 was 9.9-fold lower than that in the wild-type strain grown in low Mg^2+^ (Fig. 6A). We found that β-galactosidase activity in an E. coli wild-type strain (BW25113) harboring pYS1031 in low Mg^2+^ was 13.3-, 11.1-, and 8.4-fold higher than that in the isogenic Δ*phoP*, Δ*slyA*, and Δ*emrR* mutants, respectively (Fig. 6B). *Salmonella* EmrR protein functioned effectively in E. coli because reduced transcription of the *pagC* promoter-controlled *lacZ* fusion in the Δ*emrR* mutant was fully recovered by *emrR* overexpression from plasmid p*emrR-FLAG* (Fig. S5B). In a manner similar to the phenotype in the *Salmonella* Δ*slyA* mutant, *emrR* overexpression from p*emrR-FLAG* could also partially stimulate *lacZ* transcription in the E. coli Δ*slyA* mutant in which SlyA was absent (Fig. S5B). Furthermore, this EmrR-stimulated transcription in the E. coli Δ*slyA* mutant was reduced again by adding dopamine (Fig. S5B). Additionally, β-galactosidase activity from a Yersinia pestis wild-type strain (KIM6+) harboring pYS1031 grown in a pH 6.0 medium at 26°C was 7.9-, 8.1-, and 7.7-fold higher than that of the isogenic Δ*phoP*, Δ*rovA* (*rovA* is the *slyA* homolog in *Yersinia*; see reference 42), and Δ*emrR* mutants, respectively (Fig. 6C). These observations suggested that the same regulatory circuit constructed by these regulators should be conserved in E. coli and Y. pestis and functional even for regulation of the *Salmonella*-specific *pagC* gene.

## DISCUSSION

Here, the MarR family regulator EmrR is characterized as a virulence factor of Salmonella enterica serovar Typhimurium and Yersinia pestis and able to activate transcription by acting on the AT-rich regions at the promoters at genes such as the SPI-2 genes and also many horizontally acquired genetic loci controlled by the PhoP/PhoQ and SlyA-dependent feedforward loop.

### A role of EmrR in transcription activation as a priming antirepressor to govern a sequential regulatory module.

**FIG 7:**
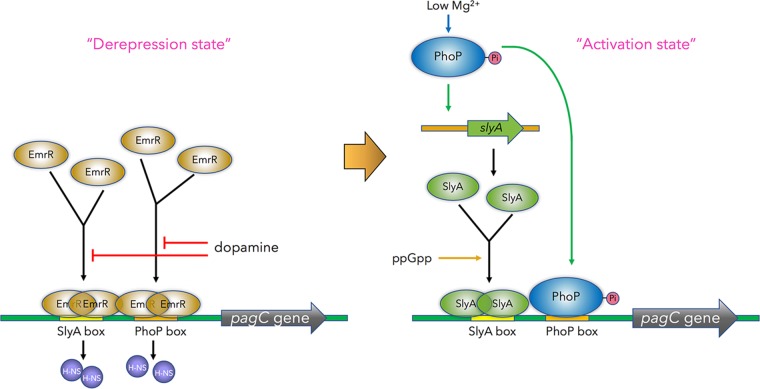
Model illustrating an EmrR-dependent gene regulation in *S.* Typhimurium. The EmrR proteins bind to the SlyA box and the PhoP box sequences through coordinate interaction to replace the transcription repressor H-NS from the *pagC* promoter region. (Left) The *pagC* promoter forms a “derepression state” after binding to EmrR. When *S.* Typhimurium is grown in low-Mg^2+^ conditions (and perhaps acidic pH or specific cationic antimicrobial peptides), the PhoP protein is phosphorylated (Pi). Transcription of the *slyA* gene is activated by the phosphorylated PhoP protein. Then, EmrR dimers in the *pagC* promoter region enhance the phosphorylated PhoP to bind the PhoP box, and also the ppGpp-bound SlyA dimer to bind the SlyA box. (Right) The PhoP- and SlyA-bound promoter further forms an “activation state” for transcription initiation. On the other hand, dopamine interacts with the EmrR protein and reduces its ability to bind the promoter region, thereby downregulating *pagC* transcription.

A well-known function of the MarR family regulators is to repress transcription mainly through exerting steric hindrance of RNA polymerase binding to target promoters by binding at the −35 and/or −10 promoter elements. Here, we have revealed an EmrR function as a transcriptional antirepressor by acting, in a manner similar to that of SlyA (5, 21, 23), on far upstream of the −35 region of the target promoters (Fig. 4B and C). In contrast with a specific direct repeat sequence recognized by SlyA (23), the EmrR binding sites lack a consensus sequence but appear to be AT-rich regions which are exemplified by the SlyA and PhoP box sequences in this study (Fig. 4C). We reason that a less sequence-specific feature will allow EmrR to interact a boarder range of the binding sites of other regulators such as SlyA and PhoP, by which it can counteract H-NS that occupies these AT-rich sequences to repress the transcription of the *ssrA* and *pagC*-*pagD* genes and many others. Initial binding of EmrR to these promoters is essential for transcription that is activated by other regulators such as SlyA and PhoP but becomes dispensable when H-NS is absent (Fig. 2E). EmrR binding to a promoter is not sufficient for transcription activation; for example, *pagC* transcription is repressed in the Δ*phoP* or Δ*slyA* mutant despite the fact that there are actually more EmrR proteins associated with the promoter (Fig. 5F). We hypothesize that the priming interaction of EmrR will make local conformation of a specific promoter, such as the *pagC-pagD* promoter, undergo a transition to form “a derepression state” (Fig. 7, left panel), which becomes more favored for subsequent binding of those successive transcription regulators. This is supported by the observation that the successive regulators SlyA and PhoP replace EmrR from the *pagC*-*pagD* promoter and also SlyA subsequently replaces EmrR from the *ssrA* promoter (Fig. 5B and G), by which the promoter region forms “an activation state” for transcription (Fig. 7, right panel). Therefore, in the Δ*slyA* mutant in which SlyA was absent, more EmrR proteins stay on both *pagC*-*pagD* and *ssrA* promoters (Fig. 5F and G), and in the Δ*phoP* mutant in which both PhoP and SlyA were absent, almost all EmrR remained on the *pagC*-*pagD* promoter (Fig. 5F). Two EmrR dimers may bind to the adjacent PhoP and SlyA boxes of the *pagC*-*pagD* promoter in a cooperative manner comparable to the λ CI dimers that bind to two neighboring operators *O_R1_* and *O_R2_* (43). This cooperative interaction must be important for EmrR binding to the *pagC*-*pagD* promoter, since nucleotide substitutions of either the SlyA box or the PhoP box were sufficient to reduce EmrR affinity dramatically to the promoter overall (Fig. 5D). The coordinate interaction between two EmrR dimers is devastated when one successive regulator displaces EmrR first, which should make it easier for the second regulator to replace another EmrR dimer from the promoter. It is worth noting that EmrR can also regulate PhoP- and SlyA-independent genetic loci, including its operon comprising *emrR*, *emrA*, and *emrB* genes encoding a multidrug resistance pump (annotated as *STM2813*, *STM2814*, and *STM2815* loci, respectively, in the *S.* Typhimurium LT2 genome). Our RNA-Seq results showed that transcription of the *emrA* and *emrB* genes in the Δ*emrR* mutant were 5.75- and 5.94-fold higher than those in the wild-type strain (see Table S3 in the supplemental material), thus reconfirming that EmrR was a negative regulator of the *emrAB* operon (9). This observation should also rule out the possibility that deletion of the *emrR* gene in our Δ*emrR* mutant had a polar effect on transcription of downstream *emrA* and *emrB* genes. Otherwise, this deletion should cause a reduction in transcription of these downstream genes. In support of this conclusion, a complementing plasmid which solely contained the *emrR* coding region (p*emrR*-*FLAG*) fully recovered EmrR-dependent transcription in the Δ*emrR* mutant to wild-type levels (Fig. 2A, D, and E).

### Neurotransmitter dopamine acts on EmrR to downregulate transcription of virulence genes.

Some gut bacteria affect the host gastrointestinal and psychological health by producing and responding to particular neuroactive compounds such as dopamine, serotonin, and norepinephrine (44). Dopamine has been considered a catecholamine signaling molecule for host-pathogen communication in the gut lumen (41, 45). Here for the first time, characterization of dopamine as a signal to interact with EmrR provides evidence that enteric bacteria are able to respond to dopamine through this MarR family regulator and probably also other specific sensor proteins. It is reasonable to say that dopamine should influence the virulence of enteric bacteria through EmrR as it is a virulence factor (Fig. 1A and Fig. S1). Dopamine is a precursor molecule of norepinephrine and epinephrine, both of which are required for full virulence of Citrobacter rodentium during mammalian infection. It was shown that host neurotransmitters epinephrine and/or norepinephrine in the gut act on two bacterial adrenergic sensors QseC and QseE to increase the virulence gene expression in enterohemorrhagic E. coli and Citrobacter rodentium (46). However, dopamine does not act on QseC or QseE, and transcription of the *pagC*-*pagD* operon is not regulated by these sensor kinases under all tested growth conditions (unpublished data). Therefore, our findings reveal the first bacterial signal transduction pathway in response to dopamine. Our study also illustrates that dopamine acts as a negative signal through sensor EmrR to inhibit transcription of specific *S.* Typhimurium genetic loci, including the SPI-2 genes and a set of divergent operons that are essential for bacterial pathogenesis. In support of its negative effect in these genetic loci, we found that dopamine reduced the ability of *Salmonella* to survive within macrophages and resistance to antimicrobial peptide (Fig. 1B and C), both of which were required for *Salmonella* virulence (28, 47). Also, several previous studies have shown that dopamine was able to decrease early *S.* Typhimurium uptake into Peyer’s patches where invasive bacteria could penetrate the specialized epithelial M cells to initiate their murine infection (48, 49). We reason that in such a host environment experienced by *Salmonella*, dopamine will act on specific sensor protein(s) to downregulate expression of those genes required for bacterial invasion. Thus, the establishment of a link between dopamine and EmrR should provide insights into molecular mechanisms to highlight how EmrR regulates *Salmonella* virulence genes and how dopamine modulates the action of EmrR in specific niches. Particularly in this study, we also provide a singular example of signal transduction requiring the action of EmrR, in which this regulator senses the level of negative signal dopamine to modulate a regulatory circuit stimulated by two positive signals, environmental low Mg^2+^ through the PhoP/PhoQ system and cytoplasmic ppGpp level through SlyA in Enterobacteriaceae.

## MATERIALS AND METHODS

### Bacterial strains, growth conditions, and oligonucleotides.

Bacterial strains used in this study are listed in Table S1 in the supplemental material. All Salmonella enterica serovar Typhimurium strains were derived from the wild-type strain ATCC 14028s (14028). All Escherichia coli strains were derived from the wild-type strain BW25113. *Salmonella* and E. coli cells were grown at 37°C in Luria-Bertani (LB) broth (Difco) or N minimal medium (50) (pH 7.4) supplemented with 0.1% Casamino Acids and 38 mM glycerol. MgCl_2_ was added to the broth or medium. All Yersinia pestis strains were derived from the wild-type strain KIM6+ (Pgm+). Bacterial cells were grown routinely at 26°C on Congo red agar from glycerol stocks to confirm the pigmentation (Pgm) phenotype of Y. pestis strains (51) and then grown on tryptose blood agar (TBA) and heart infusion broth (HIB) (Difco) (52). When necessary, antibiotics were added to bacterial cultures at final concentrations of 50 μg/ml for ampicillin (Ap), 20 μg/ml for chloramphenicol (Cm), or 50 μg/ml for kanamycin (Km). E. coli DH5α and BL21-Gold(DE3) were used as hosts for the preparation of plasmid DNA and protein production, respectively. Oligonucleotides used in this study are given in Table S2.

10.1128/mBio.02772-18.6 TABLE S1 Bacterial strains and plasmids used in this study. Download Table S1, PDF file, 0.08 MB. Copyright © 2019 Yang et al. 2019 Yang et al. This content is distributed under the terms of the Creative Commons Attribution 4.0 International license.

10.1128/mBio.02772-18.7 TABLE S2 Primers used in this study. Download Table S2, PDF file, 0.03 MB. Copyright © 2019 Yang et al. 2019 Yang et al. This content is distributed under the terms of the Creative Commons Attribution 4.0 International license.

10.1128/mBio.02772-18.8 TABLE S3 RNA-Seq analysis of the EmrR-regulated genes in *S.* Typhimurium. Download Table S3, XLSX file, 0.1 MB. Copyright © 2019 Yang et al. 2019 Yang et al. This content is distributed under the terms of the Creative Commons Attribution 4.0 International license.

10.1128/mBio.02772-18.9 TABLE S4 RNA-Seq analysis of the SlyA-regulated genes in *S.* Typhimurium. Download Table S4, XLSX file, 0.1 MB. Copyright © 2019 Yang et al. 2019 Yang et al. This content is distributed under the terms of the Creative Commons Attribution 4.0 International license.

### Construction of strains with chromosomal mutations, harboring *lac* gene fusions, or epitope-tagged proteins.

*S.* Typhimurium and *Yersinia* strains harboring deletions were generated as described previously (53). If needed, the antibiotic resistance cassette was removed using plasmid pCP20. In *Salmonella*, deletions of the *emrR*, *STM2920*, *STM1100*, *marR*, *STM1547*, *ugtL*, and *ssaM* genes were carried out using primer pairs 1857 and 1858, 2535 and 2536, 2537 and 2538, 1880 and 1881, 4478 and 4479, 2624 and 2625, and 3475 and 3476, respectively, to amplify the chloramphenicol resistance (Cm^r^) or kanamycin resistance (Km^r^) cassette from plasmid pKD3 or pKD4 (53). The PCR products were electroporated into wild-type cells harboring pKD46, and Cm^r^ or Km^r^ colonies were selected. Integration of the drug resistance cassette into the chromosomes in these mutants was confirmed by colony PCR. To construct the *Salmonella emrR*-*FLAG* strain (YS16035), a PCR fragment was synthesized with primers 1907 and 1858 from pKD3 and then electroporated into the wild type harboring pKD46, and Cm^r^ colonies were selected. The FLAG fusion was confirmed using colony PCR and DNA sequencing. Similarly, in Y. pestis, deletions of the *emrR* and *rovA* genes were carried out using primer pair 4480 and 4481 and primer pair 4482 and 4483 to amplify the Cm^r^ cassette from pKD3; the PCR products were electroporated into Y. pestis KIM6+ containing pKD46 (34). The Δ*emrR*::Cm^r^ and Δ*rovA*::Cm^r^ mutants were verified by colony PCR, and the Cm^r^ cassette was removed by using pCP20.

### Plasmid construction.

All plasmids used in this study are listed in Table S1. Plasmid p*emrR-FLAG* was constructed using PCR fragments containing the *emrR* coding region generated with primers 1910 and 1911, and *S.* Typhimurium wild-type chromosomal DNA as a template that was digested with BamHI and HindIII and then ligated between the BamHI and HindIII sites of plasmid pUHE21-2*lacI*^q^ (54). Plasmid pYS2015 was constructed using PCR fragments containing the *emrR* coding region generated with primers 1863 and 1864, and wild-type chromosomal DNA as the template that was digested with NcoI and HindIII and then ligated between the NcoI and HindIII sites of pET28a (Novagen).

### Stranded RNA-Seq analysis to collect transcriptomic data.

Total RNAs were isolated from wild-type and Δ*emrR* strains using SV Total RNA Isolation System (Promega) according to the manufacturer’s instructions from bacterial culture. Total RNAs were ribo-depleted using a Ribo-Zero rRNA removal kit (Illumina). The ribo-depleted RNAs were then enzymatically sheared to roughly 350 bp in size using KAPA’s stranded RNA-Seq kit (Roche). KAPA’s stranded RNA-Seq kit along with Illumina-compatible adapters (IDT) was used for the remaining library construction. The adapter-ligated molecules were cleaned using AMPure beads (Agencourt Bioscience/Beckman Coulter) and amplified with Kapa’s HIFI enzyme. Each library was then analyzed for fragment size on an Agilent Tapestation and quantified by qPCR (KAPA) on Quantstudio 5 (Thermo Fisher Scientific) before multiplex pooling and sequencing on a 2x75 flow cell on the NextSeq500 platform (Illumina) at the Arizona State University Genomics Core facility. RNA-Seq reads for each sample were quality checked using FastQC v0.10.1 and aligned to the *S.* Typhimurium LT2 genome from NCBI (https://www.ncbi.nlm.nih.gov/nuccore/ NC_003197.2) by TopHat v2.0.9. RNA-Seq data analysis was performed by the bacterially specific tool Rockhopper (https://cs.wellesley.edu/~btjaden/Rockhopper/index.html), including alignment, upper quartile normalization, reference-based and novel transcript identification, abundance quantification, differential gene expression detection, and operon prediction.

### Reverse transcription-PCR (RT-PCR).

Bacterial cells were grown for 4 h in N medium supplemented with 0.01 mM MgCl_2_. Dopamine (1 mM) was added when needed. Total RNAs were isolated from the bacterial culture using SV Total RNA Isolation System (Promega) according to the manufacturer’s instructions. The concentration of RNA was determined by spectrophotometry at 260 nm. The quality of RNA was confirmed by agarose gel electrophoresis. cDNA was synthesized using murine leukemia virus reverse transcriptase and random primers (New England Biolabs). DNA was amplified with primers 2031 and 2628 for *emrR*, primers 2529 and 2530 for *rpoD*, primers 2035 and 2036 for *phoP*, 2037 and 2038 for *slyA*, 3959 and 3960 for *ssaD*, 2732 and 2738 for *ssaB*, 3484 and 3485 for *sseB*, and 3447 and 3448 for *ssaM* (Table S2) using *Taq* polymerase (New England Biolabs) and performed in a thermocycler (Bio-Rad).

### *S.* Typhimurium survival assays inside J774.2 cells.

Macrophages were plated at 5 × 10^5^ cells per well in a 24-well plate and incubated overnight at 37°C in 5% CO_2_. They were then infected with stationary-phase bacteria at 5 × 10^6^ CFU in DMEM supplemented with 10% FCS and incubated for 30 min. The wells were washed three times with PBS and incubated with DMEM, 10% FCS, and 100 μg/ml gentamicin for 2 h (2 h postinfection). Bacterial invasion was determined at this time point by counting the number of CFU of the intracellular *S.* Typhimurium cells recovered from lysed macrophages in three wells infected by each bacterial strain. Then, the medium was replaced with DMEM, 10% FCS, and 12 μg/ml gentamicin, and the plate was incubated for another 16 h for bacterial cell counting. Dopamine was added to the DMEM medium at concentrations of 0.2 and 1.0 mM, or dopamine was not added. Heterologous expression of *emrR* from complementing plasmid p*emrR*-*FLAG* was induced by adding 0.2 mM IPTG. After washing the wells three times with PBS, macrophages in the wells were lysed with PBS containing 1% Triton X-100. Recovered bacterial cells were counted after grown on LB plates overnight at 37°C.

### β-Galactosidase assay.

The β-Galactosidase assay was carried out in triplicate; overnight cultures were diluted with N medium and grown for 4 h, and the activity was determined as described previously (55). Mg^2+^ was added to 0.01 mM (namely, low Mg^2+^), and 10 mM (high Mg^2+^). Complementation was carried out by using complementing plasmid p*emrR*-*FLAG,* and heterologous expression of *emrR* from this plasmid was induced by adding 0.2 mM IPTG or indicated specifically. When needed, salicylate (Sigma-Aldrich) or dopamine (Alfa Aesar, Thermo Fisher Scientific) was added to the required concentrations. Data correspond to three independent assays conducted in duplicate, and all values were means ± standard deviations.

### Expression of the *pagC* gene in the presence of phenolic compounds.

The β-galactosidase activity of the *pagC*-*lacZY* strain was determined after bacterial cells were grown for 4 h in N medium in low Mg^2+^ (0.01 mM) supplemented with or without a natural phenolic compound selected from a published list (39) and picked up from our storage. These phenolic compounds were dissolved in ethanol, DMSO, methanol, or water by following the instructions from their manufacturers and suppliers (Sigma-Aldrich, Fisher Scientific, and VWR). We chose a final concentration of these compounds for this test that did not cause a loss of bacterial viability during growth. Data correspond to three independent cultures, and all values were means ± standard deviations.

### Isolation of the EmrR-His_6_ and SlyA-His_6_ proteins.

E. coli BL21-Gold(DE3) harboring plasmids pET28a-*EmrR-his_6_* (pYS2017) and pET28a-*slyA-his_6_* (pYS1277) was grown at 37°C with shaking to an OD_600_ of 0.5 in 500 ml of LB medium; then IPTG (final concentration, 1 mM) was added, and bacteria were incubated for 2 h. Cells were harvested, washed with PBS once, resuspended in 10 ml of PBS, and lysed by sonication. The whole-cell lysates were used for EmrR-His_6_ and SlyA-His_6_ purification by mixing with His-Select Nickel Affinity Gel (Qiagen) following the instructions from the manufacturer. Pure EmrR-His_6_ and SlyA-His_6_ samples were tested using silver staining (Pierce) following the instructions from the manufacturer.

### Electrophoretic mobility shift assay (EMSA).

Primers were labeled using T4 polynucleotide kinase (New England Biolabs) and [γ-^32^P]ATP (PerkinElmer Life Sciences). ^32^P-labeled DNA fragments (10 nmol) containing *pagC*-*pagD* promoter regions, amplified by PCR from *S.* Typhimurium chromosomes with primer 522 and ^32^P-labeled primer 523 (23), were incubated at room temperature for 30 min with 0 or 50 pmol of EmrR-His_6_ and SlyA-His_6_ regulator proteins in 20 μl of an EMSA buffer (56), respectively. When required, dopamine (final concentrations of 1, 10, and 100 μM) and cold PCR products (20 nM) were added to reduce regulator protein-promoter binding and compete for the binding of regulator protein, respectively. After the addition of the DNA dye solution, the mixture was directly subjected to 4% polyacrylamide electrophoresis. Signals were detected by autoradiography.

### DNase I protection assay.

DNase I protection assays were performed using DNA fragments amplified by PCR using chromosomal DNA as the template. Before PCR, primer 523 was labeled with T4 polynucleotide kinase and [γ-^32^P]ATP. The *pagC* promoter region was synthesized with primers 522 and ^32^P-labeled primer 523. Approximately 25 pmol of labeled DNA and 0, 50, 100, or 200 pmol of the EmrR-His_6_ protein were used in a 100-μl reaction mixture. DNase I digestion was conducted as described previously (57) using 0.05 U of DNase I (Invitrogen) per reaction mixture. Samples were analyzed by 6% denaturing polyacrylamide electrophoresis by comparison with a DNA sequence ladder generated with the appropriate primer by Maxam and Gilbert A+G reaction. The positions of radioactive DNA fragments in the gels were detected by autoradiography.

### Immunoblot assay.

Bacteria producing epitope-tagged proteins were inoculated into N minimal medium (pH 7.4) with 10 mM MgCl_2_ (high Mg^2+^) for overnight growth at 37°C with aeration, and the cultures were shifted to N minimal medium (pH 7.4) with 10 μM MgCl_2_ (low Mg^2+^) and grown for 4 h. Bacterial cells were harvested and lysed by sonication. The protein concentration of whole-cell lysates was determined by using the BCA Protein Assay kit (Pierce). Protein samples were separated by 15% SDS-PAGE and transferred to nitrocellulose membranes (Amersham), and the PhoP-HA, SlyA-FLAG, and EmrR-FLAG proteins were monitored with ECL Western blotting substrate (Amersham) after incubation with monoclonal anti-HA or anti-FLAG M2 antibody (Sigma-Aldrich).

### Chromatin immunoprecipitation assay.

*S.* Typhimurium strains harboring chromosomally encoded PhoP, SlyA, and EmrR proteins with a C-terminal HA or FLAG epitope were grown in 25 ml N medium as described above. Bacterial cultures were incubated for 4 h or an indicated time. After culturing, bacterial cells were washed once with PBS and suspended in 25 ml PBS. Proteins were cross-linked to promoter DNA by adding formaldehyde to a 1% ﬁnal concentration. Chromatin immunoprecipitation (ChIP) assays were performed as described previously (58). Enriched DNA fragments containing the *pagC* promoter region were detected by PCR using primer pair 522 and 523.

### Bacterial virulence analysis in mice.

All animal procedures were approved by the Arizona State University Animal Care and Use Committee. Single colonies of each *S.* Typhimurium strain from LB agar plates were used to inoculate LB broth and grown overnight at 37°C. Bacteria were diluted (1:20) with 50 ml of fresh LB and grown to an OD_600_ of 0.85. The cells were then harvested and suspended in 1 ml PBS with gelatin. Female 7-week-old BALB/c mice from The Jackson Laboratory were inoculated by oral administration or intraperitoneal injection with bacterial suspension. Actual numbers of CFU inoculated were determined by plating serial dilutions onto LB. Mice were observed over a 30-day period to record survival. To determine the virulence of Y. pestis, single colonies of each strain from Congo red agar plates were used to inoculate HIB broth and grown overnight at 26°C. To maintain plasmid pCD1Ap, ampicillin was added to the medium at a concentration of 25 μg/ml. Bacteria were diluted with 10 ml of fresh HIB enriched with 0.2% xylose and 2.5 mM CaCl_2_ to obtain an OD_600_ of 0.1 and incubated at 26°C for subcutaneous (s.c.) infection (bubonic plague) or at 37°C for intranasal (i.n.) infection (pneumonic plague). Both cultures were grown to an OD_600_ of 0.6. The cells were then harvested and suspended in 1 ml PBS. Female 7-week-old Swiss Webster mice from Charles River Laboratories (Wilmington, MA) were inoculated by s.c. injection under the skin on the back of the neck with 100 μl of bacterial suspension or by the i.n. route with 20 μl of bacterial suspension (around 10 μl/nare). Actual numbers of CFU inoculated were determined by plating serial dilutions onto TBA. Mice were observed over a 21-day period to record survival. Data are expressed as means ± SDs. The log rank test was used for analysis of the survival curves. A *P* value of <0.05 was considered significant.

### Antimicrobial peptide killing assay.

The antimicrobial peptide susceptibility assay was described previously (59). Briefly, bacterial cultures were grown in N medium (pH 7.4) with 10 μM MgCl_2_ for 4 h. Dopamine was added to the N medium at concentrations of 0, 1.5, and 2.0 mM. Heterologous expression of *emrR* from the complementing plasmid p*emrR*-*FLAG* was induced by adding 0.2 mM IPTG. Then, bacterial cells were diluted to 1 × 10^5^ to 2 × 10^5^ ml^−1^ in fresh N medium. Five microliters of serially diluted polymyxin B (Sigma-Aldrich) was mixed with 45 μl of the bacterial suspension in each well of a 96-well plate (Cell Culture Cluster; Costar) and incubated at 37°C for 1 h. Bacterial mixtures were diluted and transferred to LB agar plates, and the plates were incubated overnight at 37°C. The number of CFU on the plates was counted. The percentage of survival was calculated as follows: survival (%) = (CFU of peptide-treated culture/CFU of no-peptide culture) × 100.
