# Supplementary figures and images for: A Dopamine-Responsive Signal Transduction Controls Transcription of Salmonella enterica Serovar Typhimurium Virulence Genes
Source: mBio. 2019 Apr 16;10(2):e02772-18. doi: 10.1128/mBio.02772-18 (PMC6469979; doi:10.1128/mBio.02772-18)

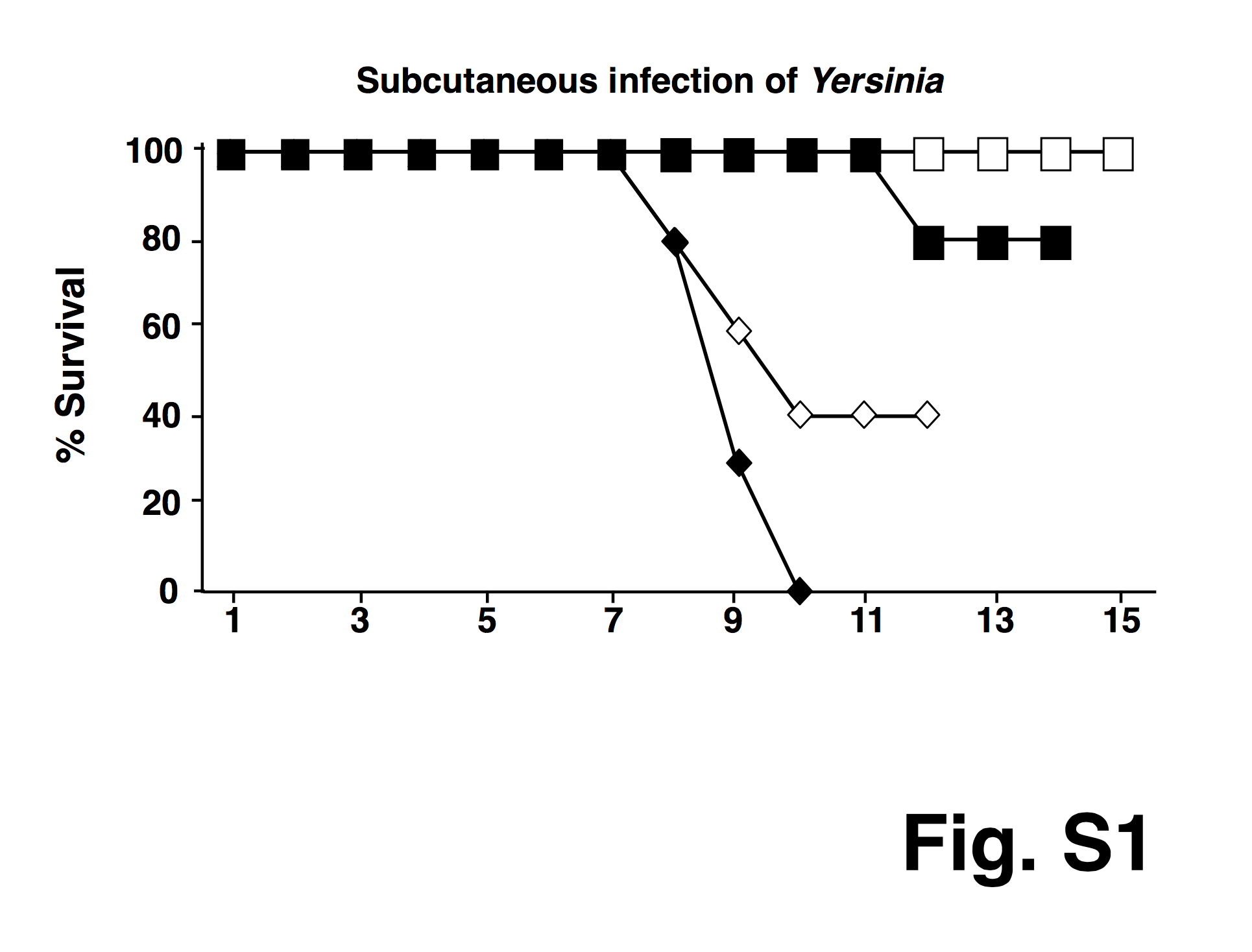

Supplement: FIG S1 [file mBio.02772-18-sf001.tif]

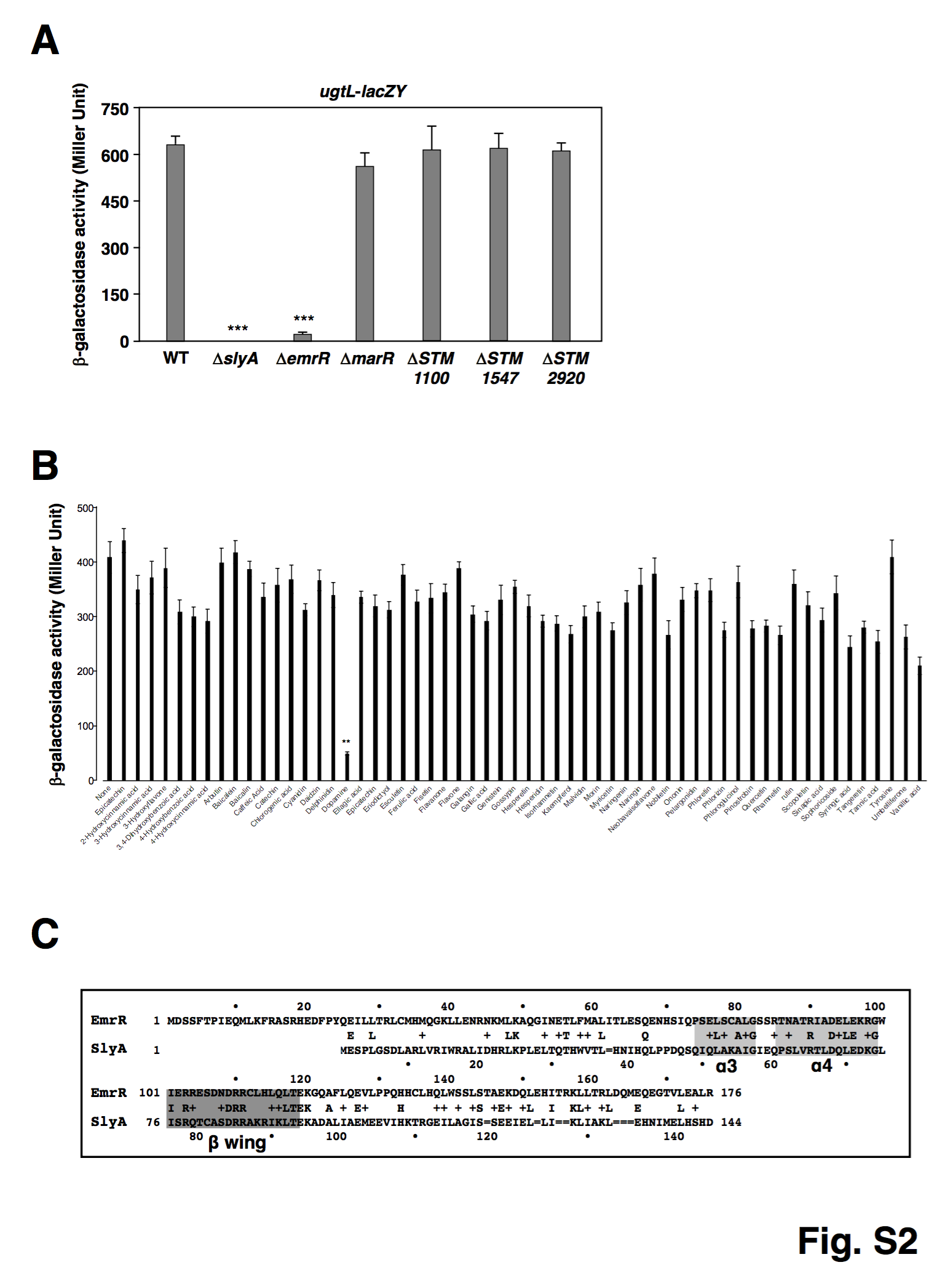

Supplement: FIG S2 [file mBio.02772-18-sf002.tif]

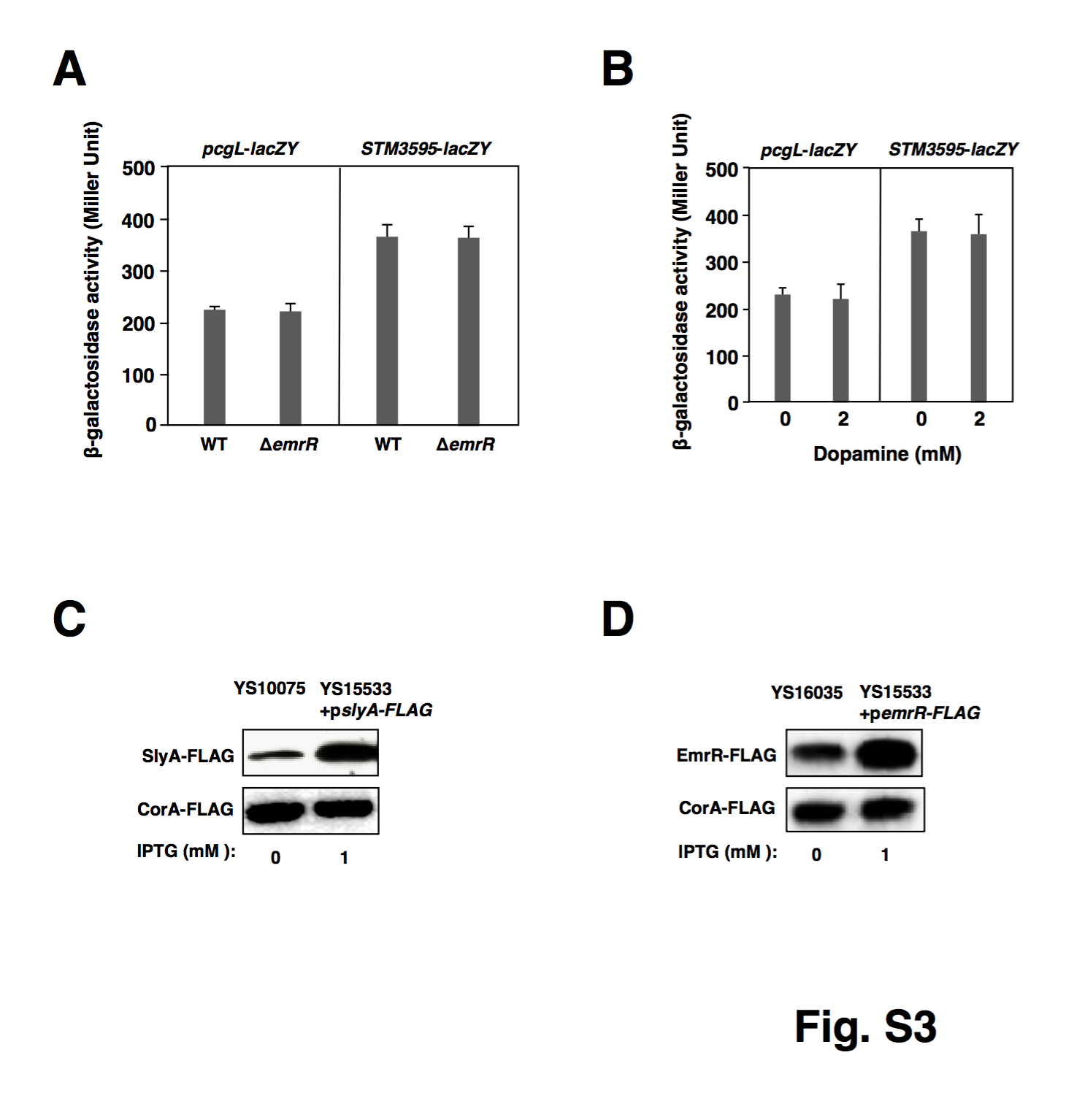

Supplement: FIG S3 [file mBio.02772-18-sf003.tif]

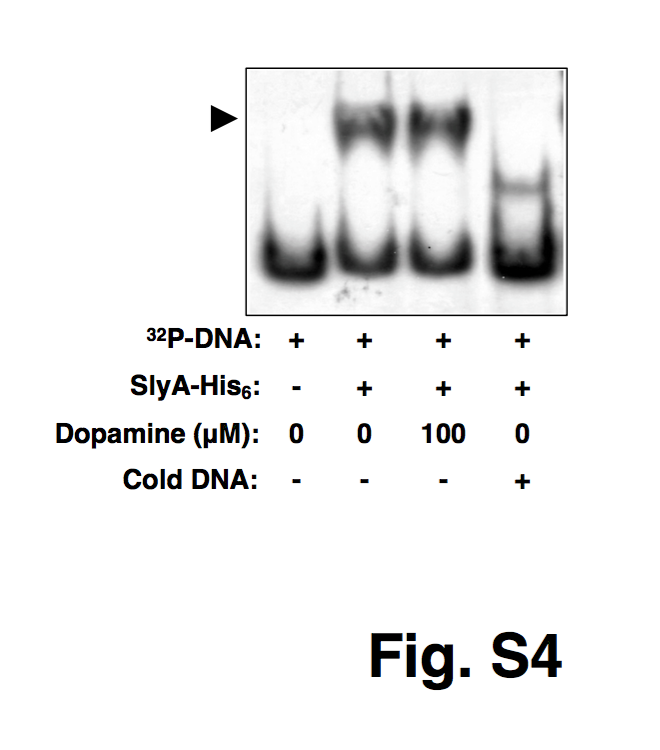

Supplement: FIG S4 [file mBio.02772-18-sf004.tif]

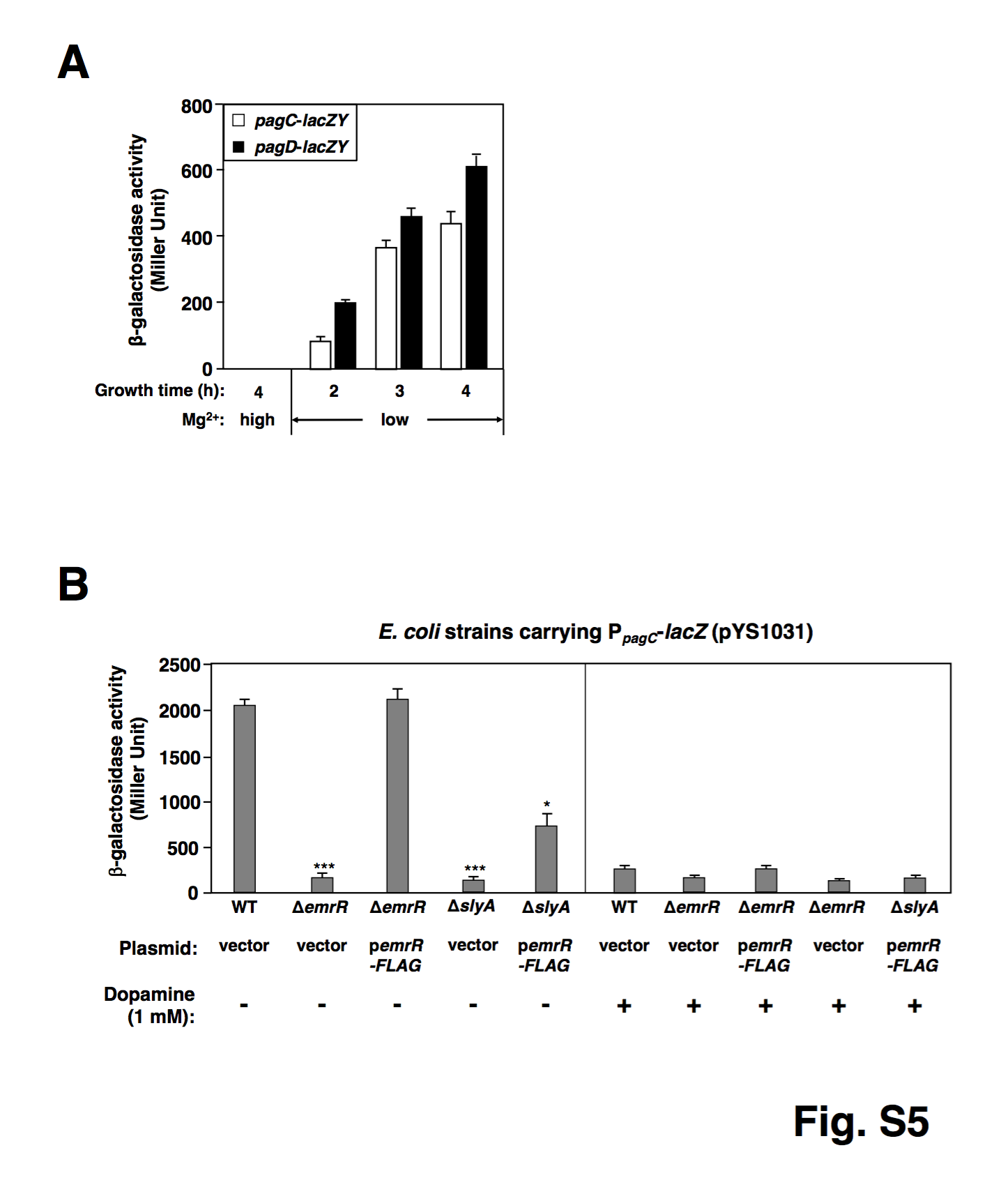

Supplement: FIG S5 [file mBio.02772-18-sf005.tif]
