# Supplementary material for: A Dopamine-Responsive Signal Transduction Controls Transcription of Salmonella enterica Serovar Typhimurium Virulence Genes
Source: mBio. 2019 Apr 16;10(2):e02772-18. doi: 10.1128/mBio.02772-18 (PMC6469979; doi:10.1128/mBio.02772-18)
Supplement: TABLE S1 [file mBio.02772-18-st001.pdf]

Table S1. Bacterial strains and plasmids used in this study

Strain or plasmid	Description	Reference or source*
S. enterica serovar Typhimurium		
14028s	wild-type	ATCC
YS15776	Δ*emrR*::Cm^R	This work
YS11590	Δ*phoP*	(26)
YS11068	Δ*slyA*	(26)
YS11708	Δ*hns*	(26)
YS15534	Δ*slyA* Δ*emrR*	This work
YS11644	*pagC-lacZY*::Km^R	(26)
YS12000	*pagD-lacZY*::Km^R	(22)
YS15451	*pagC-lacZY*::Km^R Δ*slyA*	(26)
YS14827	*pagC-lacZY*::Km^R Δ*emrR*	This work
YS11782	*pagC-lacZY*::Km^R Δ*phoP*	(26)
YS15533	*pagC-lacZY*::Km^R Δ*emrR* Δ*slyA*	This work
YS15468	*pagC-lacZY*::Km^R Δ*emrR* Δ*hns*	This work
YS17260	*pagD-lacZY*::Km^R Δ*emrR*	This work
YS17261	*pagD-lacZY*::Km^R Δ*emrR* Δ*hns*	This work
YS10382	*pcgL-lacZY*::Km^R	(26)
YS15733	*pcgL-lacZY*::Km^R Δ*emrR*	This work

YS11620	*STM3595-lacZY*::Km^R	(35)
YS15732	*STM3595-lacZY*::Km^R Δ*emrR*	This work
YS15550	*ugtL-lacZY*::Km^R	This study
YS15708	*ugtL-lacZ*::Km^R Δ*emrR*	This work
YS11610	*ugtL-lacZ*::Km^R Δ*slyA*	This work
YS15308	*emrR-lacZY*::Km^R	This work
YS15311	*emrR-lacZY*::Km^R Δ*phoP*	This work
YS18490	*ssaM-lacZY*::Km^R	This study
YS18520	*ssaM-lacZY*::Km^R Δ*emrR*	This study
YS10075	*slyA-FLAG*	(35)
YS16035	*emrR-FLAG*::Cm^R	This work
YS11591	*phoP-HA*	(35)
YS11477	*corA-FLAG*::Cm^R	(35)
YS15537	*emrR-FLAG*::Cm^R Δ*slyA*	This work
YS15538	*emrR-FLAG*::Cm^R Δ*phoP*	This work
YS15601	*phoP-HA*::Cm^R Δ*emrR*	This work
YS15602	*slyA-FLAG*::Cm^R Δ*emrR*	This work
YS17213	*emrR-FLAG*::Cm^R $SlyA\text{-}box^{-}_{pagC\text{-}pagD}$	This work
YS17214	*emrR-FLAG*::Cm^R $PhoP\text{-}box^{-}_{pagC\text{-}pagD}$	This work
YS17169	Δ*STM1100*::Cm^R	This work
YS17239	Δ*STM2920*::Cm^R	This work
YS17170	Δ*STM1547*::Km^R	This work

YS17185	Δ*marR*::Cm^R	This work
YS18662	*ugtL-lacZ*::Km^R Δ*STM1100*	This work
YS18661	*ugtL-lacZ*::Km^R Δ*STM2920*	This work
YS18660	*ugtL-lacZ*::Km^R Δ*STM1547*	This work
YS18663	*ugtL-lacZ*::Km^R Δ*marR*	This work
E. coli		
DH5α	F^- *sup*E44 Δ*lac*U169 (ϕ80 *lacZ* Δ*M15*) *hsdR*17 *recA*1 *endA*1 *gyrA*96 *thi*-1 *relA*1	(57)
BL21 (DE3)	F^- *ompT* $hsdS_B$ (r_B^- m_B^-) *gal dcm* (DE3)	(58)
BW25113	F^-DE(*araD-araB*)567*lac*Z4787 (Δ)::*rrnB-3*,LAM^-*rph-1* DE(*rhaD-rhaB*)568 *hsdR*514	ATCC
YS14986	Δ*emrR*::Km^R	(59)
YS14985	Δ*phoP*::Km^R	(59)
YS14989	Δ*slyA*::Km^R	(59)
Yersinia pestis		
Wild-type KIM6+	Pgm+, Lcr-, pMT1, (pPCP1)	(48)
χ10038	*phoP*Δ*127-429*	(60)
χ10046	Δ*rovA352*	This work
χ10064	Δ*emrR6513*	This work

Plasmids		
pKD3	rep_{R6K} γAp^{R} FRT Cm^{R} FRT	(61)
pKD4	rep_{R6K} γAp^{R} FRT Km^{R} FRT	(61)
pKD46	$rep_{pSC101}{}^{ts}$ Ap^{R} P_{araBAD} γ β exo	(61)
pCP20	$rep_{pSC101}{}^{ts}$ Ap^{R} Cm^{R} *cI*857 λP_{R}	(62)
pKG137	$rep_{R6K}\gamma$ Km^{R} FRT *lacZY* t_{his}	(63)
pUHE21-2*lac*^{q}	rep_{pMB1} Ap^{R} *lacI*^{q}	(51)
pET28a	rep_{ColE1} Km^{R} *lacI* P_{T7}	(Novagen)
pYS1031	$rep_{p15A}Cm^{R}$ $P_{pagC\text{-}wt}$ *lacZ* t_{his}	(22)
pYS1109/p*slyA-FLAG*	rep_{pMB1} Ap^{R} *lacI*^{q} *slyA-FLAG*	(35)
pYS2015/p*emrR-FLAG*	rep_{pMB1} Ap^{R} *lacI*^{q} *emrR-FLAG*	This work
pYS2017	rep_{ColE1} Km^{R} *lacI* P_{T7} *emrR-his*$_{6}$	This work
pYS1277	rep_{ColE1} Km^{R} *lacI* P_{T7} *slyA-his*$_{6}$	(22)
