# Supplementary material for: A Dopamine-Responsive Signal Transduction Controls Transcription of Salmonella enterica Serovar Typhimurium Virulence Genes
Source: mBio. 2019 Apr 16;10(2):e02772-18. doi: 10.1128/mBio.02772-18 (PMC6469979; doi:10.1128/mBio.02772-18)
Supplement: TABLE S2 [file mBio.02772-18-st002.pdf]

**Table S2. Primers used in this study***

| Primer No. | Sequence |
|---|---|
| 1857 | atc ggc tga aat taa tga ggt cat gcc caa cat atg aat atc ctc ctt ag |
| 1858 | tta atc tgg att ttt gag cgg gtc ggc gct gtg tag gct gga gct gct tc |
| 1863 | cat gcc atg gat agt tcg ttt acg ccc |
| 1864 | ccc aag ctt gcg cag cgc ctc aag aac |
| 1880 | cga aat taa tta ctt gcc ggg gca acc att cat atg aat atc ctc ctt ag |
| 1881 | cgt ctg gac atc gtc ata cct ctt ttt tgt gtg tag gct gga gct gct tc |
| 1907 | cag gaa ggc act gtt ctt gag gcg ctg cgc gac tac aag gac gac gat gac aag taa cat atg aat atc ctc ctt ag |
| 1910 | cgg gat cca atg agg tca tgc cca aat g |
| 1911 | ccc aag ccgcgtgccggcctgtccgc |
| 2031 | ggg gta ccg gat agt tcg ttt acg cc |
| 2035 | cgc cac cac ctg aag |
| 2036 | cag cct tcg cgc gcg g |
| 2037 | ggc acg gtt ggt gcg |
| 2038 | gat cgc tgg cgc agg |
| 2529 | agc agt gca aaa tgc cga ag |
| 2530 | tcc gac cac ggt ttg ttc at |
| 2535 | tga tag aaa aag tta att tat ccg gag cgc tcc ata tga ata tcc tcc tta g |
| 2536 | tgg cca aac aat agc gaa gcg gcg caa gcc gcg tgt agg ctg gag ctg ct |

| | |
|---|---|
| 2537 | ggt att aat aat tct gca aaa gag aaa cgc ttc ata tga ata tcc tcc tta g |
| 2538 | tcc cgt gtc ggc ggc gtg gga agg taa gaa tag tgt agg ctg gag ctg ctt c |
| 2624 | taa aat tat aaa aac ctg cga gga ggc tca aat aaa att ata aaa acc tgc gag gag gct caa a |
| 2625 | tat tca gta aga ccg cag gtt gca gcg gcg gag tgt agg ctg gag ctg ctt c |
| 2628 | cgg ggt acc ctga ata ctg tgg ttt tcc tg |
| 2732 | gct ggc agt ttt aaa agg c |
| 2738 | aag gct tag gta agc ttt cc |
| 3447 | ctg gcg gca agg aca ata tg |
| 3448 | tga gga ata ccc tgg aac gc |
| 3475 | ttg gct ttc tgg ctc atc atg agg cgt cag gaa cat atg aat atc ctc ctt ag |
| 3476 | ctg agc cac tgt tgc gcc ctg act ccc tca cgt gta ggc tgg agc tgc ttc |
| 3484 | ata gct tcg gcg tca gca ac |
| 3485 | ggc acg ttc agc gtt ctt ct |
| 3959 | gtc acg ttt tac aag gcc |
| 3960 | cta act ctg gct ttc ccg |
| 4478 | atg tgt att atg cac ata aga gga gac atg ctc ata tga ata tcc tcc tta g |
| 4479 | gac gat cgg att tga ccg ccc ttt tct ttt gct gtg tag gct gga gct gct tc |
| 4480 | cat tat tag taa cta tcg tta ctg tac cat cat atg aat atc ctc ctt ag |
| 4481 | tgg ttt gtt ctc tgg ata gaa ata gtg gaa gtg tag gct gga gct gct tc |
| 4482 | tat gct agc acg cta att aaa agg agg agc aac ata tga ata tcc tcc tta g |
| 4483 | tgc tgt gaa taa agt ctt tga act tta aat tag tgt agg ctg gag ctg ctt c |

*All oligonucleotides were purchased from IDT (Integrated DNA Technologies)
